# Diabetes impairs cardioprotective function of endothelial progenitor cell-derived extracellular vesicles via H3K9Ac inhibition

**DOI:** 10.7150/thno.70821

**Published:** 2022-05-21

**Authors:** Grace Huang, Zhongjian Cheng, Alycia Hildebrand, Chunlin Wang, Maria Cimini, Rajika Roy, Anna Maria Lucchese, Cindy Benedict, Vandana Mallaredy, Ajit Magadum, Darukeshwara Joladarashi, Charan Thej, Carolina Gonzalez, May Trungcao, Venkata Naga Srikanth Garikipati, John W. Elrod, Walter J. Koch, Raj Kishore

**Affiliations:** 1 Center for Translational Medicine, Lewis Katz School of Medicine, Temple University, Philadelphia, PA, 19140; 2 Department of Emergency Medicine, The Ohio State University Wexner Medical Center, Columbus, OH 43210; 3 Dorothy M Davis Heart and Lung Research Institute, The Ohio State University Wexner Medical Center, Columbus, OH 43210; 4 Department of Cardiovascular Sciences, Lewis Katz School of Medicine, Temple University, Philadelphia, PA 19140

**Keywords:** Cardiac injury, Endothelial progenitors, Extracellular vesicle, Histone acetylation, Angiogenesis

## Abstract

**Background and Purpose:** Myocardial infarction (MI) in diabetic patients results in higher mortality and morbidity. We and others have previously shown that bone marrow-endothelial progenitor cells (EPCs) promote cardiac neovascularization and attenuate ischemic injury. Lately, small extracellular vesicles (EVs) have emerged as major paracrine effectors mediating the benefits of stem cell therapy. Modest clinical outcomes of autologous cell-based therapies suggest diabetes-induced EPC dysfunction and may also reflect their EV derivatives. Moreover, studies suggest that post-translational histone modifications promote diabetes-induced vascular dysfunctions. Therefore, we tested the hypothesis that diabetic EPC-EVs may lose their post-injury cardiac reparative function by modulating histone modification in endothelial cells (ECs).

**Methods:** We collected EVs from the culture medium of EPCs isolated from non-diabetic (db/+) and diabetic (db/db) mice and examined their effects on recipient ECs and cardiomyocytes *in vitro,* and their reparative function in permanent ligation of left anterior descending (LAD) coronary artery and ischemia/reperfusion (I/R) myocardial ischemic injuries *in vivo*.

**Results:** Compared to db/+ EPC-EVs, db/db EPC-EVs promoted EC and cardiomyocyte apoptosis and repressed tube-forming capacity of ECs. *In vivo*, db/db EPC-EVs depressed cardiac function, reduced capillary density, and increased fibrosis compared to db/+ EPC-EV treatments after MI. Moreover, in the I/R MI model, db/+ EPC-EV-mediated acute cardio-protection was lost with db/db EPC-EVs, and db/db EPC-EVs increased immune cell infiltration, infarct area, and plasma cardiac troponin-I. Mechanistically, histone 3 lysine 9 acetylation (H3K9Ac) was significantly decreased in cardiac ECs treated with db/db EPC-EVs compared to db/+ EPC-EVs. The H3K9Ac chromatin immunoprecipitation sequencing (ChIP-Seq) results further revealed that db/db EPC-EVs reduced H3K9Ac level on angiogenic, cell survival, and proliferative genes in cardiac ECs. We found that the histone deacetylase (HDAC) inhibitor, valproic acid (VPA), partly restored diabetic EPC-EV-impaired H3K9Ac levels, tube formation and viability of ECs, and enhanced cell survival and proliferative genes, *Pdgfd* and *Sox12*, expression. Moreover, we observed that VPA treatment improved db/db EPC-mediated post-MI cardiac repair and functions.

**Conclusions:** Our findings unravel that diabetes impairs EPC-EV reparative function in the ischemic heart, at least partially, through HDACs-mediated H3K9Ac downregulation leading to transcriptional suppression of angiogenic, proliferative and cell survival genes in recipient cardiac ECs. Thus, HDAC inhibitors may potentially be used to restore the function of diabetic EPC and other stem cells for autologous cell therapy applications.

## Introduction

Cardiovascular disease (CVD) causes higher mortality and disability in patients with diabetes and imposes a significant health and economic burden 1. Over the past several decades, accumulating preclinical evidence has shown that bone marrow-derived endothelial progenitor cells (EPCs) participate in neovascularization and ischemic myocardial protection and repair 2, 3. This led to the autologous EPC-based cardiac clinical trials, which although safe, demonstrated only modest functional benefits 4, 5. For the autologous EPC and/or other progenitor cell-based therapy after myocardial infarction (MI), their quality and functional potency depends on their origin and may be compromised by existing co-morbidities such as diabetes-induced cell dysfunction. Previous studies have indicated that hyperglycemia, the primary risk factor of diabetes, induces EPC dysfunction 6, 7. Clinical evidence further shows that EPCs isolated from type I and II diabetic patients have decreased mobilization and homing, proliferation, and angiogenic capacity, suggesting diabetic/hyperglycemic insult dampens EPC reparative and angiogenic functions in ischemic cardiac injury 8.

In recent years, emerging consensus suggests that adult stem/progenitor cells benefit myocardial repair and regeneration majorly through paracrine mechanisms. Small extracellular vesicles (EVs), of less than 150 nm in diameter (also termed as exosomes) constitute a major functional paracrine entity of stem cells and carry a variety of bioactive molecules cargo such as proteins, small RNAs, and lipids that alter recipient cell behaviors^9^. In this perspective, stem/progenitor cell-derived EV therapy has been put forward as an alternative cell-free modality for ischemic myocardial repair with encouraging results in the cardiac injury models of both small and large animal models 10, 11. However, it is also being recognized that EV production, their cargo constituents and their functional activity depend largely on the pathophysiological environment of their parental cells 12. Our previous studies have shown that external stimuli/stress such as inflammation and hyperglycemia changes EPC/stem cell-derived EV contents and functional properties 13, 14. In addition, EVs from diabetic plasma have been shown to be deficient in protecting cardiomyocytes 15. However, whether EPCs isolated from diabetic mice exhibit functionally impaired EVs and the underlying mechanism for diabetic-EPC EV dysfunction is not well established.

Epigenetic dysregulations including DNA methylation and histone modifications result in altered gene expression contributing to diabetic complications 16, 17. Commonly described epigenetic modifications include post-translational modifications of histone tails, such as methylation and acetylation of specific lysine residues. These modifications shift chromatin patterns from a compact (heterochromatin) to an open (euchromatin) structure or *vice versa*, enabling gene repression or activation depending on the accessibility of chromatin 18. Histone H3 Lysine 9 (H3K9) modifications (both acetylation and methylation) were reported to be involved in inflammatory phenotype in the vascular smooth muscle cells in diabetes 19. Specifically, hyperglycemia is crucial in mediating histone modification enzyme cooperation, which leads to epigenetic mark changes 20. However, whether EVs from diabetic EPCs/stem cells are involved in histone modifications and consequent gene expression changes in recipient non-diabetic cells is not well established.

This study demonstrates that diabetes deteriorates EPC-EV reparative function in myocardial injuries post permanent LAD ligation (MI) and ischemia-reperfusion (I/R), by decreasing angiogenesis and cardiac functions, increasing maladaptive remodeling, infarct area, cardiomyocyte death and immune cell infiltration. Mechanistically, we show that diabetic EPC-EVs enriched with HDAC1 protein, downregulate the gene activation mark, histone 3 lysine 9 acetylation (H3K9Ac), through increasing histone deacetylase (HDAC) enzymatic activity, which leads to a decrease in a multitude of endothelial functional gene repression in recipient mouse cardiac endothelial cells (MCECs). Furthermore, diabetic EPC-EV-mediated H3K9Ac reduction can be reversed by pretreatment of donor diabetic EPCs with HDAC inhibitor valproic acid (VPA). These findings suggest that diabetes impairs EPC-EV reparative activities in ischemic myocardium. Mechanistically, we report that diabetic EPC-EVs worsen cardiac repair partly through HDAC activity-H3K9Ac-mediated cell survival/proliferative gene inhibition in ECs.

## Methods

All data presented in this study are freely available from the corresponding author upon any reasonable request. The Online Data Supplement provides additional detailed materials and methods.

### Vertebrate animals, cell isolation and culture

All animal procedures were performed following the approved protocols of the Institutional Animal Care and Use Committee of Temple University. Ten to twelve-week-old male mice (C57BL/6J) were purchased from Jackson Research Laboratory (Bar Harbor, ME) for MI surgery. Eight to ten-week-old diabetic male mice (#000642 BKS.Cg-*Dock7^m^* +/+ *Lepr^db^*/J-homozygous) hereafter db/db and nondiabetic mice (#000642 BKS.Cg-*Dock7^m^* +/+ *Lepr^db^*/J-heterozygous) hereafter db/+ were purchased from Jackson Research Laboratory for EPC isolation described in our previous paper^21^. EPCs were isolated from mouse marrow of tibiae, femurs and hip bones and were cultured in endothelial cell basal medium‐2 (EBM‐2, Clonetics) supplemented with kit (EGM-2 SingleQuote, Clonetics) and 10% EV-depleted FBS (Thermo Fisher Scientific, A2720801). After 4 days, db/+ EPCs were cultured at 37°C, 5% CO_2_ atmosphere. Male mice were used to avoid the reproductive hormone effect in female mice. Human CD34+ Hematopoietic stem cells (HSC) were purchased from AllCells, LLC and were cultured in a human hematopoietic cell culture medium (StemSpan^TM^ SFEMII, STEMCELL) supplemented with 10X CD34+ expansion supplement (STEMCELL, #02691) and 0.25% human albumin (Sigma, A9731). HSCs were cultured in normal glucose media or in high glucose (25 mM) media in 37°C, 5% CO_2_ atmosphere.

### EV isolation and characterization

EV collection, purification, storage, and identification were performed as previously described 22. Detailed characterization of EPC-EV was described in our previous papers 13, 23. In brief, media from diabetic and db/+ EPCs were collected daily and then concentrated for the 30% sucrose gradient ultracentrifugation EV isolation method. EVs were dissolved in PBS and aliquoted for long-term storage at -80°C. EV particle numbers were measured by Nanosight (NS300, Malvern) using Nanoparticle Tracking Analysis (NTA) software.

### Nanoparticle Tracking Analysis (NTA)

NS-300 Nanosight instrument (Malvern Instruments Ltd, Malvern, UK) equipped with a sCMOS camera (Hamamatsu Photonics, Hamamatsu, Japan) and a 405 nm laser was used. Data acquisition and processing were performed using NTA software version 2.3 build 0025. Background extraction was applied, and automatic settings were employed to determine the minimum expected particle size, minimum track length, and blur settings. Data were obtained at camera level 12 (shutter: 600, gain: 350). Three movies of 30 s at 25 frames per second were recorded and assigned a single measurement in triplicates.

### Induction of MI models

The ligation of the left anterior descending (LAD) coronary artery was performed as a permeant MI model described previously 13. A detailed description of methods for LAD ligation and Ischemia/reperfusion models are provided in supplementary methods.

### Echocardiography

Transthoracic two-dimensional M-mode echocardiography using the Vevo2100 equipped with 30 MHz transducers (VisualSonics, Toronto, ON, Canada) was performed before MI (baseline), and 1-, 2-, 3-, and 4- weeks after surgery as described previously 24. Mice were anesthetized with a mixture of 1.5% isoflurane and oxygen (1 L/min) with an isoflurane delivery system (Viking Medical, Medford, NJ). The internal diameter of the LV was measured in the short-axis view from M-mode recordings; percent ejection fraction (% EF) and fractional shortening (% FS) were calculated using corresponding formulas as previously described 25.

### Plasma cardiac troponin-I measurement

Mouse blood was collected from vena cava after 24 hours I/R and immediately centrifuged at 4000 rpm for 10 min at 4°C. Plasma was then collected, and cTn-I concentrations were measured by kit following the manufacturer's instruction (Life Diagnostic, Inc # cTNI-1-HS).

### Tissue preparation and immunohistochemistry

Mouse heart tissue samples were fixed in 10% formalin for at least 48 hours and embedded in paraffin. Cardiac tissues were cross-sectioned into 4-5 μm-thick slides. Masson Trichrome staining (Sigma, HT15-1KT) and TUNEL staining (Roche, 12156792910) were performed following the manufacturer's instruction and previously described in detail 24, 26. For the identification of endothelial cells and pan immune cells, CD31 (R&D, AF3628), CD45 staining (R&D, AF114). Cardiomyocytes were identified by staining for α-Sarcomeric Actin (α-SARC, Sigma-Aldrich, A2172). Images were acquired using the Niko Eclipse Ti fluorescence microscope using 4X, 10X, 20X and 40X objectives and planimetry analysis using ImageJ.

### Tube formation assay

1X10^4 human or mouse microvascular endothelial cells (HMVECs and mouse cardiac endothelial cells (MCECs)) were cultured in EV-depleted culture media while treated with vehicle, 1X 10^6 db/+ EPC-EV or 1X 10^6 diabetic EPC-EV particles for 48 hours. HMVECs or MCECs were then re-plated on Matrigel (Corning, 365231) in a 48-well plate for 16 hours. Images were taken using a phase-contrast microscope to count total branch points.

### Nuclear Extraction and HDAC activity assay

1X10^6 MCECs were treated with 1X 10^9 db/+ EPC-EV, or 1X 10^9 diabetic EPC-EV particles for 24 hours. Cells were then collected, and nuclear proteins were isolated according to the manufacturer's instructions (ThermoFisher Scientific, #78840). 10 mg nuclear protein was used to measure HDAC activity according to the manufacturer's instructions (Epigentek, P-4034-48).

### RNA extraction, reverse transcription, and RT-PCR

RNA was isolated from cells using miRNeasy Mini kit (Qiagen, 1038703). NanoDrop-1000 (Thermo Scientific) was used to identify RNA concentration and purity determined by 260A/280A ratio. High-capacity cDNA Reverse Transcription Kit (Applied Biosystems, 4368814) was used to obtain cDNA. RT-PCR was performed on an Applied Biosystems 770 StepOnePlus system using the Fast SYBR^TM^ Green Master Mix (Applied Biosystems) according to the manufacturer's instructions. Fold changes were normalized to GAPDH with the threshold delta-delta cycle method. Primers are listed in Table S1.

### H3K9Ac chromatin Immunoprecipitation and sequencing (ChIP-seq)

MCECs were fixed with 1% formaldehyde for 15 min and quenched with 0.125 M glycine after db/+ or db/db EPC-EV treatment for 24 hours and then processed for ChIP-seq using the antibody against H3K9Ac (Active Motif cat# 39917). Input controls were used in normalizing CHIP-seq signals. ChIP and ChIP analysis was performed by Active Motif, Inc. Detailed methods are provided in Online Data Supplement.

### Cell lysate preparation and western blotting

All procedures were carried out as reported previously 13. Briefly, cell protein samples were collected in a lysate buffer following BCA protein quantification. Samples were run by electrophoresis on Mini-PROTEAN TGX gels (BIO-Red). All antibody information can be found in Table S2.

### Statistical analysis

Statistical analyses were performed using GraphPad Prism 7.0 software (GraphPad, La Jolla, CA). All Data are presented as mean ± SEM and represent at least 3 independent biological experiments. Unpaired t-test was used to compare 2 sample groups. For comparisons among > 2 groups, one-way ANOVA followed by Tukey multiple comparisons test was performed. For echocardiography parameters with repeated measures over time, two-way ANOVA with Tukey's multiple comparisons test was used. A *P* value of < 0.05 was considered as statistical significance.

## Results

### Diabetic EPC-EVs are functionally impaired

**Figure 1:**
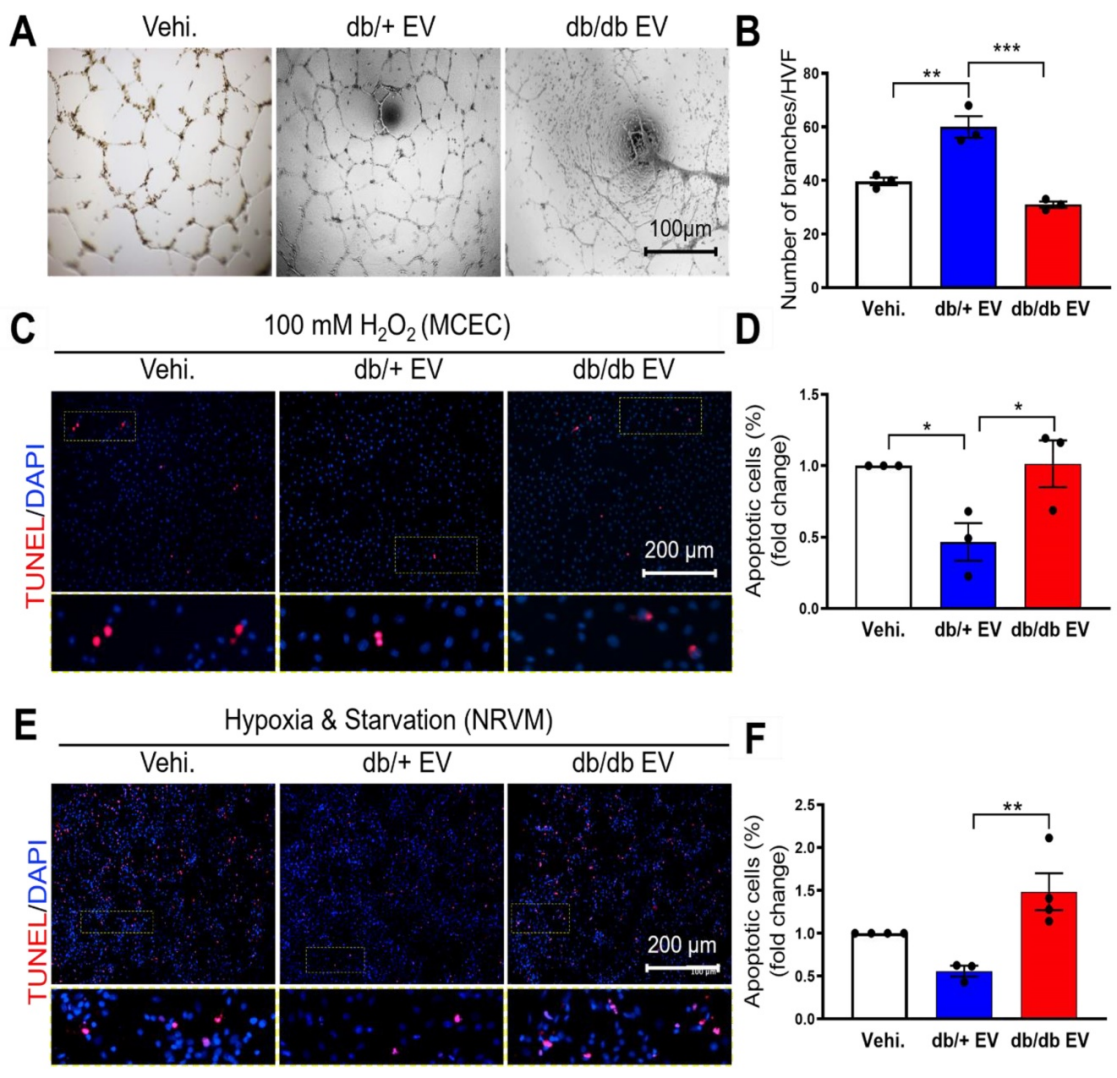
** Diabetic EPC-EVs repress endothelial cell tube formation and endothelial and cardiomyocyte cell survival.** (A). db/db EPC-EVs reduced tubulogenesis in HMVECs. (B). Quantification analysis of branch number per HVF. (C). db/db EPC-EVs failed to prevent MCEC apoptosis under H_2_O_2_ treatment. MCECs were treated with vehicle, db/+ EPC-EVs, or db/db EPC-EVs for 24 hours followed by 5-hour 100 µM H_2_O_2_ treatment. Apoptotic MCECs were indicated by TUNEL staining (red). Nuclei, DAPI (blue). Enlarged images showed the co-localization of TUNEL staining and DAPI. (D). Quantification analysis of apoptotic cells was calculated by TUNEL-stained and DAPI double-positive cells versus total cells. (E). db/db EPC-EVs failed to prevent NRVM apoptosis under CoCl_2_ treatment. NRVMs were treated with vehicle, db/+ EPC-EVs or db/db EPC-EVs for 24 hours followed by 24-hour starvation and CoCl_2_ (100 µM) treatment. Apoptotic NRVMs were indicated by TUNEL staining (red). Nuclei, DAPI (blue). Enlarged images showed the co-localization of TUNEL staining and DAPI. (F). Quantification analysis of apoptotic cells was calculated by TUNEL-stained and DAPI double-positive cells versus total cells. All Data are shown as mean ± SEM, n≥3 for each group. ***P < 0.001, **P < 0.01, *P < 0.05. DAPI, 4′,6-diamidino-2-phenylindole, EPC-EV, endothelial progenitor cell-derived extracellular vesicle; HMVEC, human microvascular endothelial cell; HVF, high visual field; MCEC, mouse cardiac endothelial cell; NRVM, neonatal rat cardiomyocyte; Vehi., vehicle.

We have previously shown that bone marrow-derived cells under diabetic conditions are functionally impaired 7. To understand whether diabetes impairs EPC-EV function and how db/db EPC-EVs lose their cardiac reparative activities, EPC-EVs were collected from 10-week-old db/+ and db/db mice. Db/db mice showed higher body weight and blood glucose level compared with db/+ mice (Figure S1A-B). EV size and yield were similar between db/+ and db/db groups (Figure S1C-E). Identity of EV was also confirmed by the protein expression of EV markers Alix and HSP70 (Figure S1F). We then examined the biological function of db/+ and db/db EPC-EVs in ECs and cardiomyocytes *in vitro*. Neovascularization/angiogenesis is crucial for cardiac repair post MI 27. Therefore, we performed tube formation assays as a measure of endothelial angiogenic activity in response to EPC-EVs and found that the EC branch number was significantly increased by db/+ EPC-EV while this tube formation activity was significantly diminished by db/db EPC-EV treatments (Figure 1A-B). Furthermore, increased oxidative stress occurs in the ischemic heart and reduces cell viability 28. Therefore, we pre-treated MCECs with db/+ EPC-EVs, db/db EPC-EVs or vehicle for 24 hours, followed by 100 µM H_2_O_2_ stress. We found that db/+ EPC-EVs enhanced MCEC survival but db/db EPC-EVs failed to provide protection from MCEC death (Figure 1C-D). Also, cardiomyocyte apoptosis is a major event after a cardiac injury 29, we determined the effect of db/+ and db/db EPC-EVs on neonatal rat ventricular myocyte (NRVM) cell death. To mimic the nutrient deprivation and hypoxic environment in the ischemic area post-MI, we incubated NRVMs with db/+ EPC-EVs, db/db EPC-EVs or vehicle for 24 hours followed by starvation and hypoxia for 48 hours. The results showed that db/+ EPC-EVs partly prevented cardiomyocyte apoptosis while db/db EPC-EVs failed to prevent myocyte apoptosis which was like vehicle-treated group (Figure 1E-F). Together, our data suggest that EVs from db/db EPCs not only lose the protection offered by db/+ EPC-EV but often worsen the recipient cell functions. We also confirmed internalization of EPC-EVs by MCECs and AC16 cardiomyocyte cells by labeling the EPC-EVs with PKH26 (Figure S2A-B).

### Diabetic EPC-EVs lose their reparative properties in a permanent LAD ligation MI model

**Figure 2:**
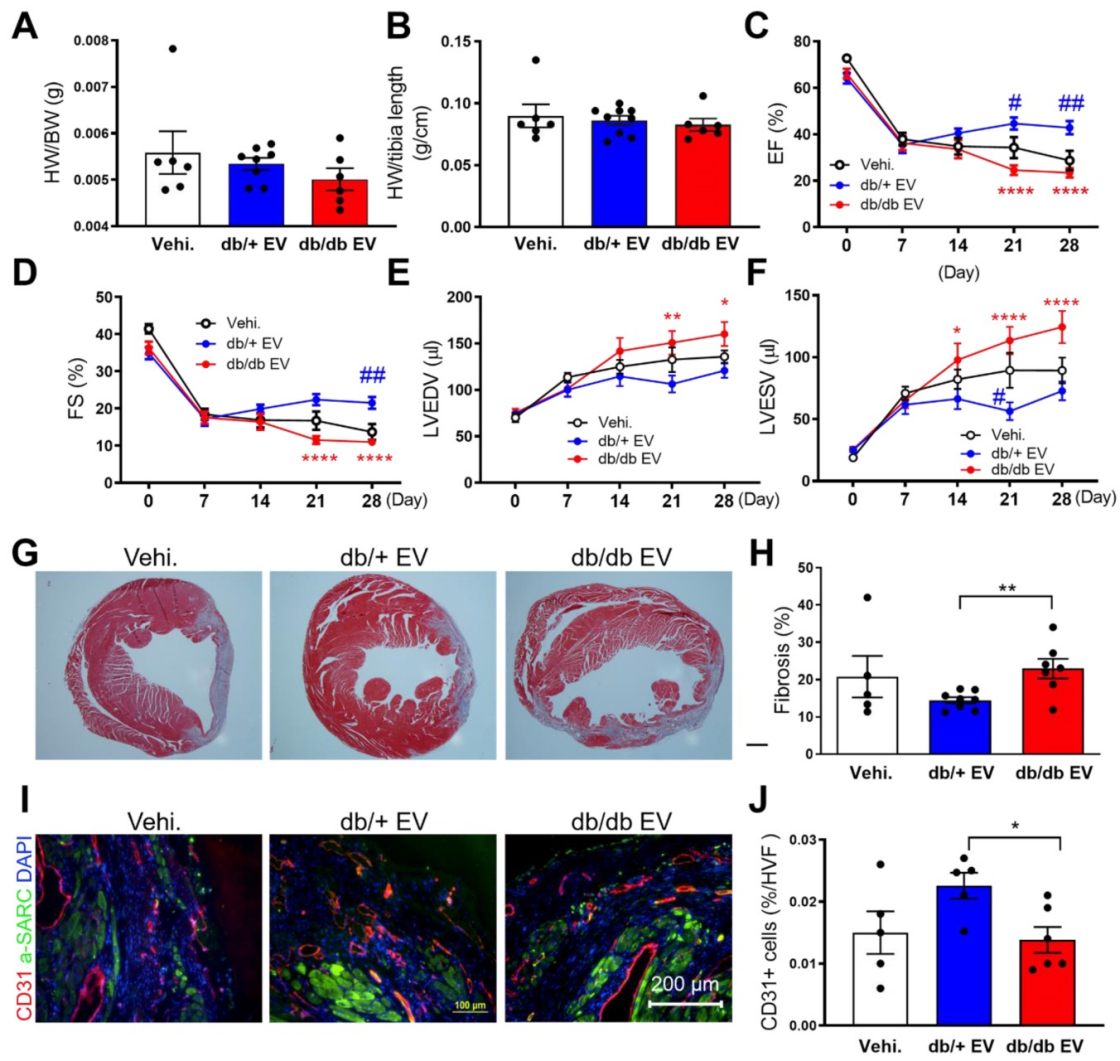
** Diabetic EPC-EV deteriorate cardiac function, remodeling and neovascularization post MI.** Baseline measurements were conducted, and mice then underwent LAD coronary artery ligation followed by vehicle, db/+ or db/db EPC-EV treatment. (A) HW/BW ratio and (B) HW/TL ratio were similar in vehicle, db/+ EPC-EV or db/db EPC-EV treated mice 1 month after MI. (C-F). Echocardiographic data showed db/db EPC-EV impaired cardiac function in MI mice. (C). EF (****P < 0.0001, *^#^P* = 0.049, *^##^P*=0.0049), (D). FS (****P < 0.0001, *^##^P* = 0.0071) (E) LVEDV (*P = 0.0120, **P = 0.0017), and (F) LVESV (*P = 0.0203, ****P < 0.0001, ^#^*P* = 0.0355) at various time points post MI for each group. *P is significantly different between db/+ EPC-EV and db/db EPC-EV; ^#^*P* is significantly different between vehicle and db/+ EPC-EV. BW, body weight; EF, ejection fraction; FS, fraction shortening; HW, heart weight; LAD, left anterior descending; LV, left ventricle; LVEDV, left ventricular end-diastolic volume; LVESV, left ventricular end-systolic volume; MI, myocardial infarction. The number of animals included in each parameter of numerical echocardiographic data is shown in Table S3. (G) Masson's trichrome-stained hearts at day 28 post MI. (H) Quantification of fibrotic area. (I). Left Ventricular cross-sections (infarct border zone) were immunolabeled with CD31 (red) and α-SARC (green) antibodies. Nuclei, DAPI (blue). (J). Planimetry analysis of capillary number by quantifying the changes of CD31 immunolabeling in border zone per HVF. n=5-13. All Data are shown as mean ± SEM. **P < 0.01. α -SARC, α-sarcomere actin; HVF, high visual field; MI, myocardial infarction; Vehi., vehicle.

To investigate the impact of db/+ and db/db EPC-EVs on cardiac function in the ischemic heart *in vivo,* db/+ and db/db EPC-EVs (1X10^9 particles) or vehicle were injected into three locations in the border zone around the ligation site. At 4 weeks after MI, db/db EPC-EV treated mice exhibited unchanged heart weight to body weight ratio (HW/BW), HW to tibia length ratio (HW/TL) compared to db/+ EPC-EV-treated mice (Figure 2A-B). Echocardiography was performed at 0 (baseline), 1-, 2-, 3- and 4-week post-MI to measure cardiac function. LV ejection fraction (EF%) and fraction shortening (FS%) were improved in db/+ EPC-EV-treated mice compared to vehicle-treated mice. However, EF and FS were significantly reduced, while LV end-systolic volume and LV end-diastolic volume were increased in db/db EPC-EV compared to db/+ EPC-EV treated mice at week 4 post-MI (Figure 2C-F). These findings suggest that db/db EPC-EV lose their beneficial effects in ischemic hearts. Of note, db/db EPC-EV dampened cardiac function more severely than vehicle group, suggesting db/db EPC-EV not only lose their beneficial effects but may trigger adverse consequences in ischemic hearts.

### Diabetic EPC-EVs promote maladaptive tissue remodeling and impair neovascularization post-MI

To investigate the effect of db/db EPC-EVs on cardiac fibrosis, Masson trichrome staining of cardiac tissue was performed. Results showed that fibrosis was markedly increased in db/db EPC-EV compared with db/+ EPC-EV group suggesting db/db EPC-EVs enhanced infarct size and scar formation (Figure 2G-H). As neovascularization/angiogenesis are essential for cardiac repair post MI 27, we performed evaluation of vessel density in the border zone of the infarct by CD31 staining, and found that CD31 positive vessels were significantly decreased in db/db EPC-EV treated mice compared with db/+ EPC-EV treated mice (Figure 2I-J). This indicates that db/db EPC-EVs impaired vessel/capillary formation in the border zone post-MI. Collectively, these data provided evidence that EVs from db/db EPCs lose their cardiac protective, anti-fibrotic and angiogenic activities of db/+ EPC-EVs.

### Non-diabetic EPC-EVs protect against ischemia-reperfusion (I/R) injury, while diabetic EPC-EVs do not provide acute cardio-protection

**Figure 3:**
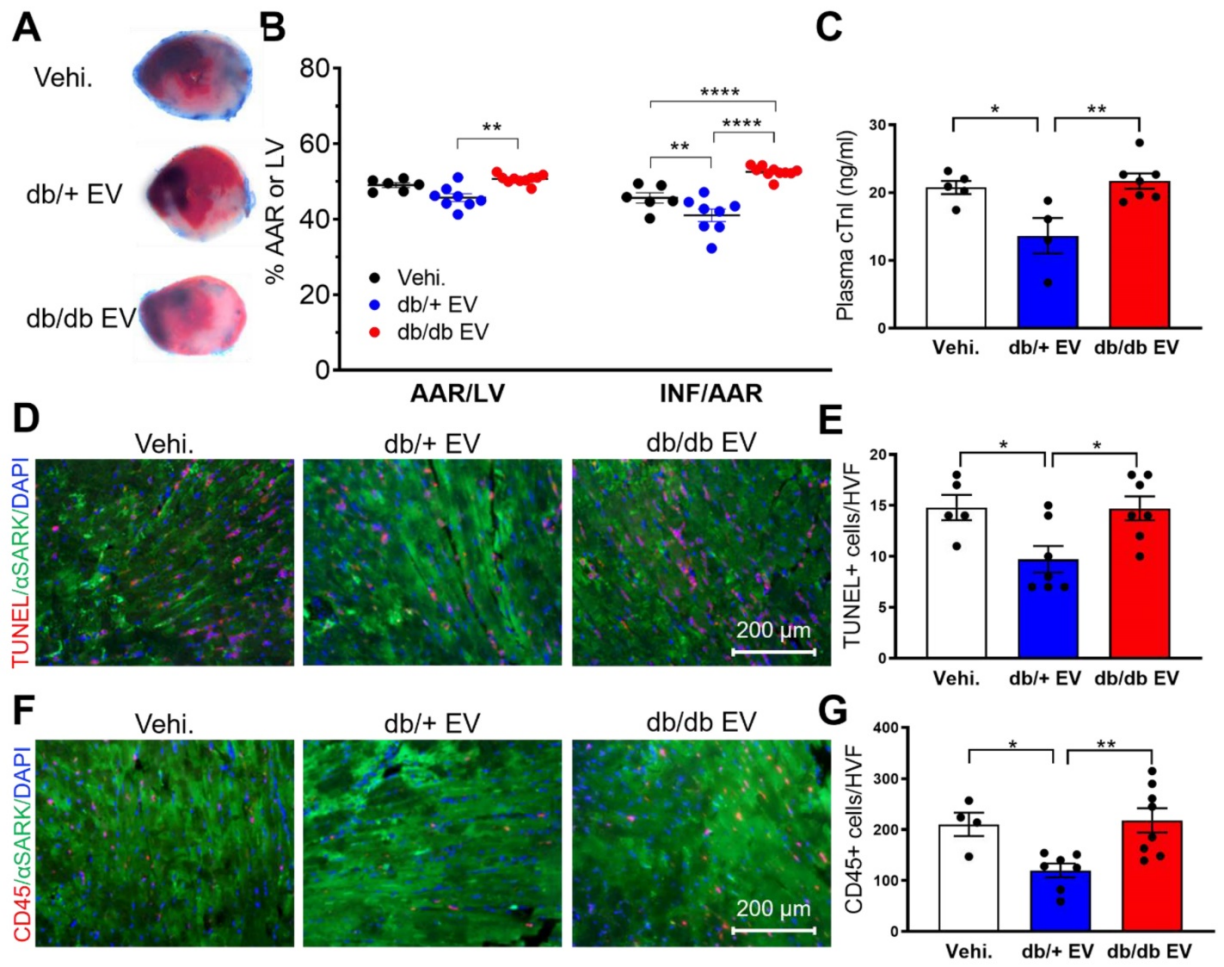
** Diabetic EPC-EVs lose protective effect against myocardial I/R injury using intramuscular injection.** (A). db/db EPC-EVs increased infarcted area in I/R mice. The secondary ventricular cross-sections of TTC-stained hearts from base to apex area. (Evan's blue-stained area, non-ischemic zone; remaining area, area-at risk; white area, infarcted tissue; red area, viable myocardium). (B). Planimetry analysis of infarct size by quantifying Evan's blue dye excluded area, AAR, LV area, and non-TTC-stained area: INF. n=6-10. (C). db/db EPC-EVs increased plasma cTn-I level in I/R mice. Plasma cTn-I was measured 24 hours after I/R using cTn-I ELISA kit. (D). db/db EPC-EVs promoted cell apoptosis in I/R mice. Apoptotic cardiomyocytes were indicated by TUNEL staining (red) and α-Sarcomeric actin (green) antibody. Nuclei, DAPI (blue). (E). Quantification analysis of apoptotic cells was calculated by TUNEL/alpha-SA-double stained and DAPI double-positive cells versus total cells. n=5-7 (F). db/db EPC-EVs promoted immune cell infiltration in I/R mice. Ventricular cross-sections were immunolabeled with pan immune cell marker CD45 (red) and α-Sarcomeric actin (green) antibody. Nuclei, DAPI (blue). (G). Quantification of immune cell infiltration per HVF. n=4-8. All Data are shown as mean ± SEM. ****P < 0.0001, ***P < 0.001, **P < 0.01, *P < 0.05. AAR, area-at-risk; HVF, high visual field; INF, infarct; I/R, ischemia reperfusion; LV, left ventricle; Vehi., vehicle.

In clinical practice, timely reperfusion is pivotal for patients with cardiac ischemia. However, I/R induces the second wave of injury. To elucidate the acute cardioprotective effect of db/+ and db/db EPC-EVs on myocardium, post-I/R, we subjected mice to 45-minute ischemia, followed by 24-hour reperfusion. db/+ EPC-EV, db/db EPC-EV or vehicle were injected near the infarcted area during reperfusion by intramuscular injection. TTC staining was conducted to delineate the infarcted area and viable tissue (Figure 3A). The results showed that compared to vehicle treated mice, db/+ EPC-EVs significantly reduced infarct size (INF). However, both area at risk (AAR) and INF were significantly increased in the db/db EPC-EV treated group than db/+ EPC-EV treated group (Figure 3B). Furthermore, plasma cardiac troponin I (cTn-I) level is commonly used to diagnose cardiac injury in patients. We found that vehicle and db/db EPC-EV treatment groups showed augmented plasma cTn-I levels whereas db/+ EPC-EVs showed lower plasma cTn-I level (Figure 3C). In agreement with cell apoptosis *in vitro* (Figure 1C-F), we found an increased TUNEL positive cardiomyocytes in the I/R heart of mice treated with vehicle and db/db EPC-EV treatment compared to the mice that received db/+ EPC-EV (Figure 3D-E). Also, I/R injury evokes inflammation that leads to immune cell recruitment 30. We performed CD45 staining to examine immune cell infiltration. The results showed that vehicle and db/db EPC-EVs induced more immune cell infiltration to the ischemic area than db/+ EPC-EVs, which may trigger a more severe inflammatory response (Figure 3F-G). These studies provide convincing evidence that db/+ EPC-EVs protect against acute I/R injury, but db/db EPC-EVs are deficient in acute myocardial protection activities. Taken together, db/+ EPC-EVs but not db/db EPC-EVs showed the acute cardioprotective activities post I/R injury suggesting administration of healthy EPC-EVs as a potential therapeutic approach for acute injury of MI patients with reperfusion using the intramuscular injection strategy.

### Diabetic EPC-EVs repress H3K9Ac by increasing HDAC enzyme activity in recipient MCECs

**Figure 4:**
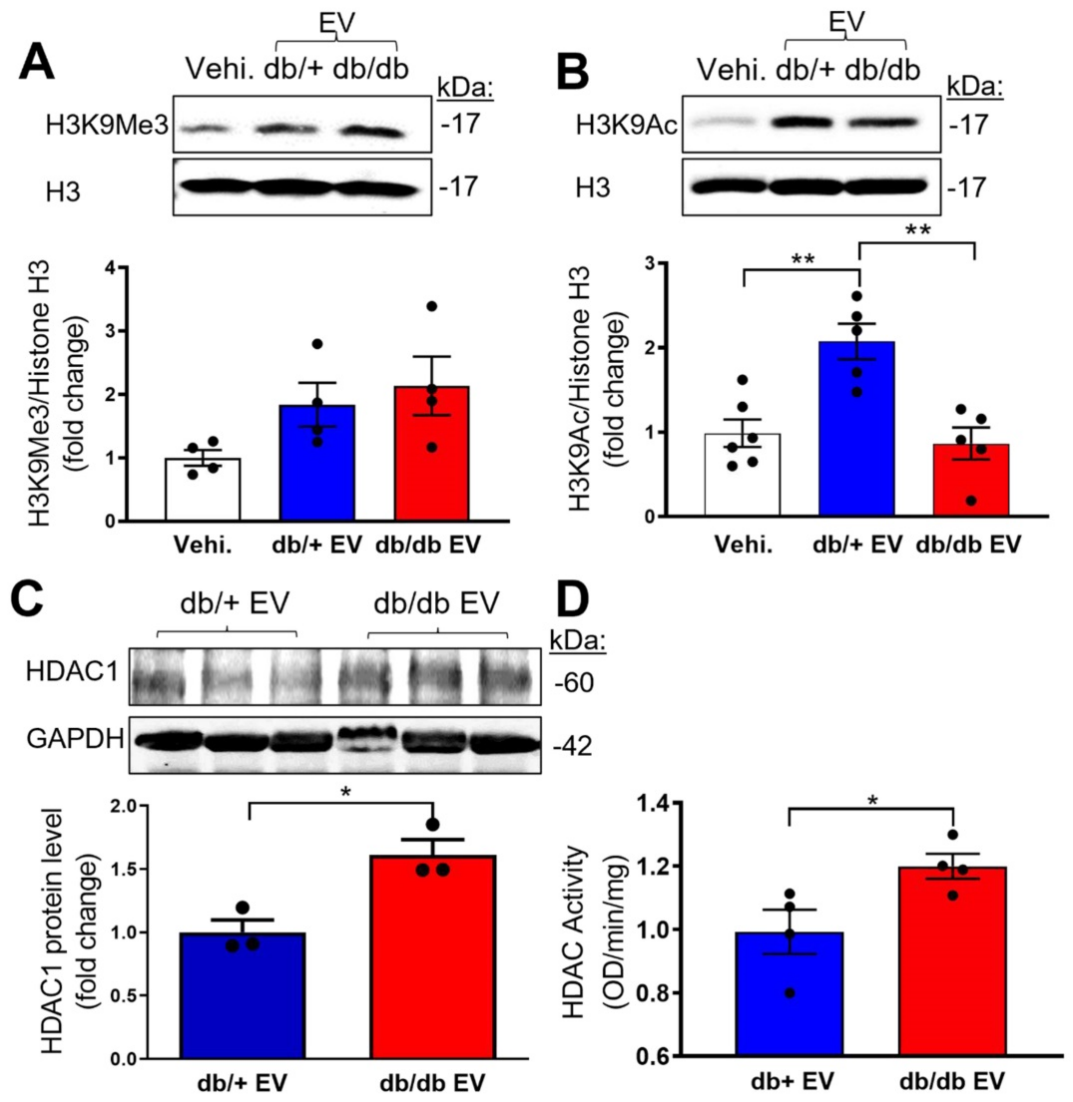
** Diabetic EPC-EVs downregulate H3K9Ac level with increase HDAC enzymatic activity in MCECs.** MCECs were treated with vehicle, db/+ or db/db EPC-EV for 24 hours. (A). db/db EPC-EVs trended but did not significantly change H3K9Me3 level in MCECs compared to vehicle and db/+ EPC-EV treated groups. (B). db/db EPC-EVs significantly decreased H3K9Ac level in MCECs compared to db/+ EPC-EV treated group. Cells were collected, lysed, and subjected to Western blotting using H3K9Me3 or H3K9Ac antibodies and quantification of protein expression. (C). db/db EPC-EVs contained higher level of HDAC1 protein. (D). db/db EPC-EVs increased HDAC activity in MCECs. Nuclear proteins were isolated, and HDAC enzymic activities were measured and results were plotted. All Data are shown as mean ± SEM. n > 3 for each group. **P < 0.01, *P < 0.05. MCEC, mouse cardiac endothelial cell; H3K9Me3, histone H3 lysine 9 trimethylation; H3K9Ac, histone H3 lysine 9 acetylation; HDAC, histone deacetylases.

Epigenetic dysregulation is well-known in diabetes 31. Specifically, histone 3 lysine 9 trimethylation (H3K9Me3) is reported as a constitutively repressive mark involved in suppressing target genes 32. To address if db/db-EPC-EVs may alter recipient EC functions via H3K9Me3-mediated endothelial gene inhibition, we treated MCECs with vehicle, db/+ EPC-EVs or db/db EPC-EVs for 24 hours. The results indicated a trending but not statistically significant difference in the expression of the gene repressive mark, H3K9Me3 by db/db EPC-EVs (Figure 4A). As the epigenetic gene regulation is also known to be modified by the active epigenetic mark of acetylation (Ac) on the same histone 3 lysine residue, H3K9Ac was examined in MCEC treated with vehicle, db/+ and db/db EPC-EVs. Interestingly, H3K9Ac level was significantly decreased under db/db EPC-EV treatment, indicating a suppressed endothelial gene transcription by the reduction in H3K9Ac (Figure 4B). Histone deacetylase (HDAC) family controls the acetylation state of lysine residues. HDAC class I members: HDAC1, HDAC2, and HDAC3 were identified as acetyl group removing enzymes and have been tested as therapeutic targets for CVD 33. To test if db/db-EPC-EVs are enriched in HDAC, we examined HDAC1 level in db/+ and db/db EPC-EVs. We observed that EPC-EVs from db/db mice contained higher protein level of HDAC1 compared to that from db/+ mice (Figure 4C), suggesting db/db EPC-EVs may transfer more HDAC1 to ECs thereby increasing HDAC activity and leading to reduction in gene activation epigenetic mark, H3K9 acetylation. We further determined HDAC1, HDAC2, and HDAC3 protein level in EPC-EVs-treated MCECs. Interestingly, we did not find significant changes in their protein expression (Figure S3A-B). HDAC enzymatic activity was significantly increased in MCECs treated with db/db EPC-EVs (Figure 4D). These findings suggest that db/db EPC-EV deliver HDAC1 to recipient MCECs thereby increasing HDAC enzymatic activity and reduction in H3K9Ac.

### Diabetic EPC-EVs decrease H3K9Ac level at transcription start site of recipient endothelial cell genes

**Figure 5:**
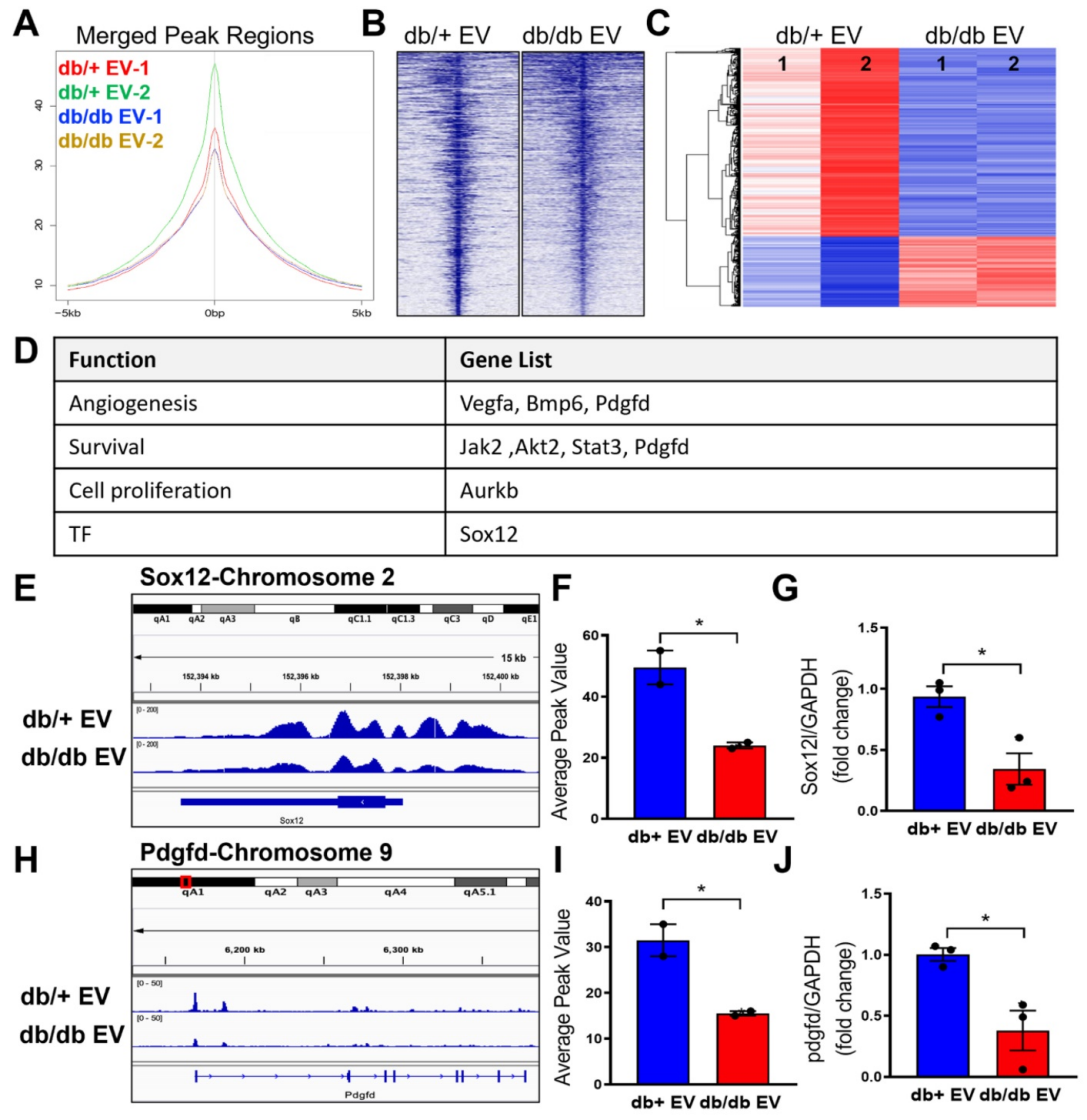
** Non-diabetic EPC-EVs increase H3K9Ac level at the transcription start site of genes in MCECs.** MCECs were treated with db/+ or db/db EPC-EVs for 24 hours and subjected to CHIP-seq against H3K9Ac. (A-B). db/db EPC-EVs decreased H3K9Ac level in MCECs. (A). Average peak values (y-axis) were plotted vs. sequencing tags across the TSS (-5000 to +5000 bp relative to the TSS; x-axis). (B). Sequencing tags distribution (y-axis) across the TSS (-5000 to +5000 bp relative to the TSS; x-axis) is presented as heatmaps. (C). The portion of differentially upregulated genes is greater in db/+ EPC-EV treated MCECs than db/db EPC-EV treated MCECs. Differentially expressed db/+ and db/db EPC-EV treated MCECs are shown as a heatmap in duplicate. n=2 for CHIP-seq experiment. (D). db/+ EPC-EV enhanced angiogenic/ cell survival/ proliferative gene expression. An upregulated gene list (FC > 2, *P* < 0.05) in functional categories. (E and H). db/db EPC-EV decreased H3K9Ac level at TSS of Sox12 and Pdgfd in MCEC. Integrative genomics viewer (IGV) software was used to view the fragment density of H3K9Ac (y-axis) aligned along with the gene coordinates (x-axis). (F and I) Data are presented as bar graphs representing the mean. n=2 for each group (G and J) db/db EPC-EV decreased Sox12 and Pdgfd expression in MCECs. Total RNA was extracted, Sox12 and Pdgfd gene expression were examined using qRT-PCR. The results were plotted. n=3 for qRT-PCR validation in each group. All Data are shown as mean ± SEM. *P<0.05. Pdgfd, platelet-derived growth factor D; Sox12, SRY-box transcription factor 12; TSS, transcription start site.

To elucidate which genes are enriched with H3K9Ac level, we further performed H3K9Ac-ChIP-seq experiments in MCEC treated with db/+ and db/db EPC-EVs. In line with the H3K9Ac protein expression, the ChIP-seq results showed the lowest percentage of H3K9Ac enrichment in the merge peak regions under db/db EPC-EV treatment compared with db/+ EPC-EV treatment, indicating db/db EPC-EV reduced chromatin accessibility for gene transcription (Figure 5A-B). Moreover, in the differentially expressed genes (FC > 2; *P* < 0.05), the portion of differentially upregulated genes (indicated in red color) of db/db EPC-EV treated MCECs is less than db/+ EPC-EV treated MCECs (Figure 5C). Among differentially upregulated genes of db/+ EPC-EV treated group, we identified beneficial genes involved in angiogenesis (Vegfa, Bmp6 and Pdgfd), cell survival (Jak2, Akt2, Stat3 and Pdgfd), and pro-proliferation (Aurkb), and transcription factor (Sox12), which is relative to cell proliferation, mobilization, and survival (Figure 5D). Representative genes, Sox12 and Pdgfd, are shown in Figure 5E-F and 5H-I and additional ChIP-seq data is shown in Figure S4 and Tables S4-5). Sox12 has decreased H3K9Ac level, upstream, and in-gene regions (upper panel); similarly, Pdgfd has decreased H3K9Ac level in gene regions (lower panel) after db/db EPC-EV treatment. As expected, quantitative PCR results confirmed a reduction in Sox12 and Pdgfd mRNA levels (Figure 5G and 5J) in response to db/db EPC-EV treatment. db/db EPC-EVs also tended to decrease H3K9Ac level at TSS of survival/proliferative gene vegfa, Bmp6, Jak2, Akt2, Stat3 and Aurkb in MCECs (Figure S4). Together, these data suggest that db/db EPC-EV may deteriorate cardiac repair through decreasing angiogenic and cell survival gene transcription by reducing H3K9Ac levels in recipient ECs. Together, these data suggest that db/+ EPC-EV may benefit cardiac repair through enhancing angiogenic and cell survival gene transcription by increasing H3K9Ac levels in recipient cells while db/db EPC-EV fail to enhance H3K9Ac levels.

### HDAC inhibitor, valproic acid (VPA) partly rescues diabetic EPC-EV-impaired H3K9Ac expression, tube formation and cell survival activities of MCECs

**Figure 6:**
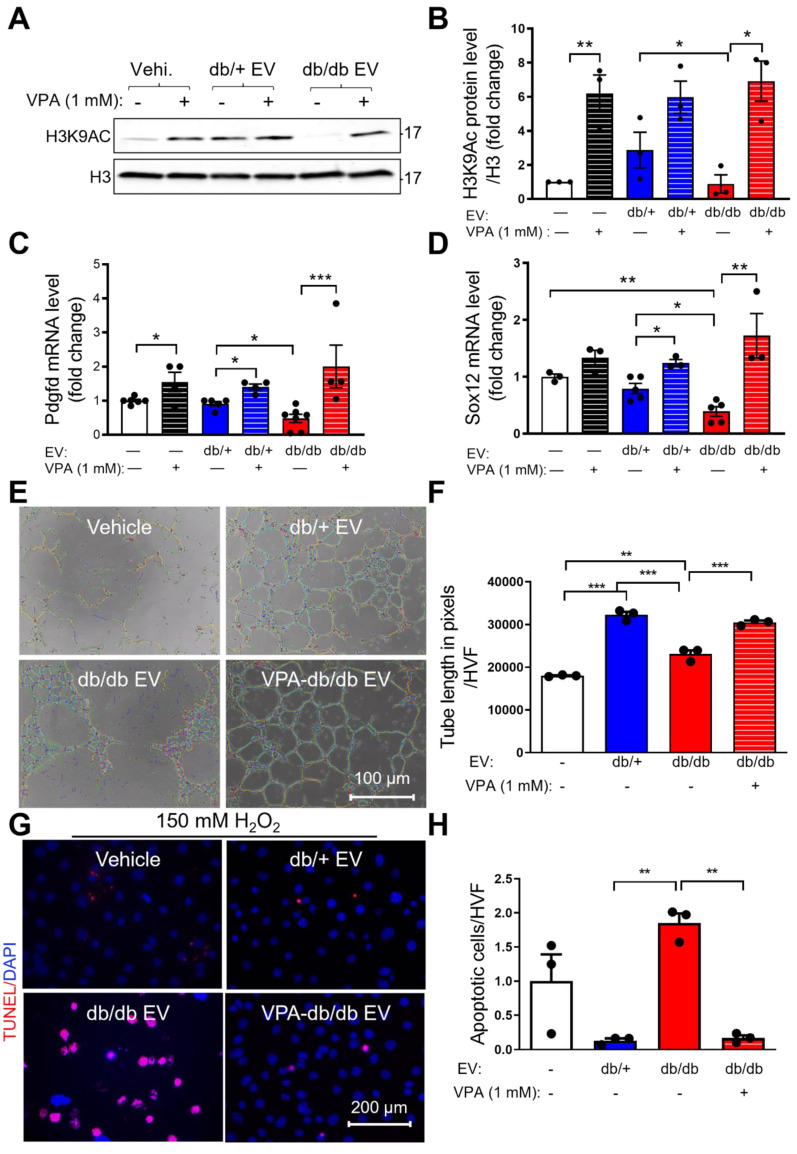
** Pan-HDAC inhibitor, valproic acid (VPA) partly rescues diabetic EPC-EV-mediated H3K9Ac and gene downregulation, and diabetic EPC-EV-impaired tube formation and cell survival.** MCECs were treated with vehicle, db/+ or db/db EPC-EVs along with or without 1 mM VPA, for 24 hours. (A and B). VPA rescued diabetic EPC-EV-mediated H3K9Ac downregulation. Cells were collected, lysed, and subjected to Western blotting using H3K9Ac antibodies and quantification of protein expression. (C and D). VPA rescued diabetic EPC-EV-mediated Pdgfd and Sox12 downregulation. Total RNA was extracted, Sox12 and Pdgfd gene expression were examined using qRT-PCR. The results were plotted. (E-F). VPA partially restored db/db EPC-EV dysfunction by improving MCEC tube formation. MCEC were treated with vehicle, db/+ EPC-EVs, db/db EPC-EVs and VPA (1 mM)-treated EPC-EVs (1 MCEC: 500 EV particles during tube formation). (G-H). VPA treatment of db/db EPCs rescued untreated db/db EPC-EV-mediated MCEC death under hypoxia condition. MCECs were treated with vehicle, db/+ EPC-EVs, db/db EPC-EVs and VPA (1 mM)-treated EPC-EVs ((1 MCEC: 1000 EV particles during tube formation) for 24 hours then exposed to 150 µM H2O2 for 6 hours)). n=3. All Data are shown as mean ± SEM. n≥3 for each group. ***P < 0.001, **P < 0.01, *P < 0.05. H3K9Ac, histone H3 lysine 9 acetylation; Pdgfd, platelet-derived growth factor D; Sox12, SRY-box transcription factor 12; VPA, valproic acid.

Recently, it was reported that the pan-HDAC inhibitor, VPA, received FDA approval for the treatment of epilepsy. It was also shown that VPA protected heart function post-AMI 34. To examine if VPA can reverse db/db EPC-EV-regulated H3K9Ac reduction, we treated MCECs with 1 mM VPA for 24h along with vehicle, db/+ or db/db EPC-EV treatments. The data showed that VPA significantly increased H3K9Ac level in the db/db EPC-EV treated group, and db/+ EPC-EV and vehicle treated groups (Figure 6A-B). Moreover, we found that VPA reversed db/db EPC-EV mediated Sox12 and Pdgfd gene downregulation (Figure 6C-D). Importantly, we observed that pretreatment of db/db EPCs with VPA significantly restored db/db EPC-EV dysfunction by improving both MCEC tube formation activity (Figure 6E-F) and enhancing protection from H_2_O_2_-induced MCEC death (Figure 6G-H). These data support the critical role of HDACs in the functional dysregulation of diabetic EPC-derived EVs.

### VPA pretreatment enhances db/db EPC cardiac reparative activity in MI model

**Figure 7:**
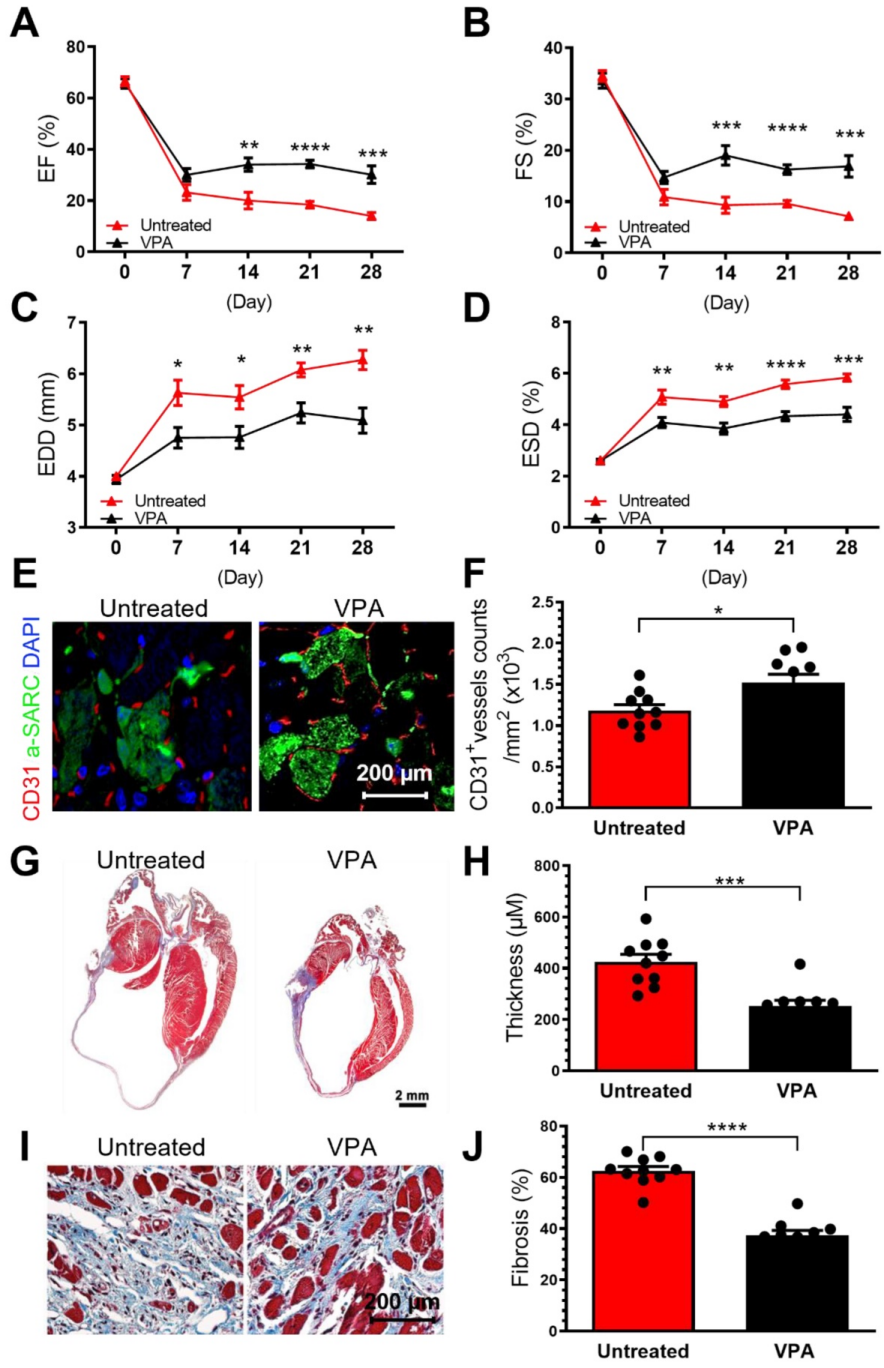
** Intracardiac transplantation of VPA-pretreated db/db EPCs improve post-MI cardiac functions.** EPCs isolated from 8-week-old male db/db mice were treated or untreated with VPA (1 mM) for 24 hours and were injected used for intracardiac injections immediately after ligation of left coronary artery (2*10^5 cells in 20 µl PBS into three locations in the border zone around the ligation site. (A-D). Echocardiographic data showed VPA-pretreatment partly restored untreated db/db EPCs-impairment in augmenting cardiac function post-MI in wildtype C57BL6/J mice. (A). EF, (B) FS, (C) EDD and (D) ESD at various time points post MI for each group. (E). VPA treated db/db-EPCs improved neovascularization in MI heart. (F). Quantification of CD31+ vessels in infarct border zone. Ventricular cross-sections were immunolabeled with CD31 (red) and α-Sarcomeric actin (green) antibody. (G). Compared to untreated db/db EPCs, VPA pretreated db/db EPCs improved LV wall thickness in MI mice. Representative images of Masson's trichrome-stained hearts at 4 weeks post MI. (H). Quantification of LV wall thickness. EF, ejection fraction; FS, fraction shortening; LV, left ventricle; EDD, end-diastolic dimensions; ESD, end-systolic dimensions. (G -J). Compared to untreated db/db EPCs, VPA pretreated db/db EPCs improved LV wall thickness (G-H) and fibrosis (I-J) in MI mice. Ventricular cross-sections were stained with Masson's trichrome. EF, ejection fraction; FS, fraction shortening; LV, left ventricle; EDD, end-diastolic dimensions; ESD, end-systolic dimensions. All Data are shown as mean ± SEM. n=10. ****P < 0.0001; ***P < 0.001; **P < 0.01; *P < 0.05.

It has been demonstrated that specific cargo of EVs, including their RNA, protein, and lipid contents, are reflective of donor cell origin and its physiological state. Db/db-EPC-EVs largely recapitulate the functional deficiency of parent db/db EPCs which we have reported be functionally impaired 7. Since VPA treatment of db/db-EPC-EV regains protective function like non-diabetic EPC EVs, we next examined whether VPA pretreatment may rescue cardiac reparative functions of db/db-EPCs in permanent ligation-MI model. We found that compared to db/db-EPCs which showed no cardiac reparative activity, intramyocardial injection of VPA-pretreated db/db EPCs significantly improved cardiac LV functions as % EF, % FS and end diastolic and end systolic dimensions (EDD, ESD) were all significantly improved (Figure 7A-D). Similarly, vascular density, and left ventricular wall thickness and fibrosis post-MI (Figure 7A-J), were also significantly enhanced by VPA-pretreated diabetic-EPCs compared to untreated diabetic EPCs indicating inhibition of HDAC activity partly reverses defective phenotype of diabetic EPCs.

### Angiogenesis capacity of hCD34+ cell-derived EVs is impaired under a hyperglycemic insult

Lastly, to test the translational aspect of mouse studies, we collected EVs from human CD34+ stem cells stimulated with 25 mM glucose, mimicking the hyperglycemic condition, which is characteristic of diabetic patients. We performed a tube formation assay in HMVECs and found that the tube branch numbers were significantly decreased in cells treated with EVs from hCD34+ cells subjected to 25 mM glucose culture conditions compared with normal hCD34+ EVs and vehicle. In line with our mouse study (Figure 1A-B), these data suggest that the angiogenesis capacity of hCD34+ cell-derived EV is also impaired under a hyperglycemic insult (Figure S5A-B).

## Discussion

In the past decades, numerous preclinical studies reported that autologous BM-EPCs repair ischemic heart by promoting cardiac neovascularization and cardiac function, along with mitigating inflammation, oxidative stress, apoptosis, and maladaptive remodeling 35 leading to BM progenitors/EPC-based clinical trials. Clinical trials of bone marrow progenitor/EPC based cell therapy in patients with CVD did not meet the expected clinical outcomes despite universally positive preclinical studies 35. This may suggest that the functionality of autologous cells obtained from the diseased patients with multiple comorbidities like diabetes may be compromised. Indeed, clinical data suggest that diabetic patients, who received autologous stem cell therapy to restore microvascular reperfusion, suffered significant major adverse cardiac events compared to non-diabetic patients 36. Our previous study has shown that diabetes impaired bone marrow angiogenic cell reparative function. In line with our findings, Natalia et al. specifically indicated that the function and yield of different EPC populations derived from peripheral blood of diabetic patients were deficient 37. Over the last several years, it has become evident that EVs secreted from stem cells largely recapitulate the functional properties of their parent cells 11-13, 15. In fact, stem cell EVs are seriously considered for clinical applications as a cell-free modality. However, since the cargo of EVs largely reflects the physical state of cells of origin, it is reasonable to assume that EVs from diabetic EPCs/stem cells may reflect functional deficiencies akin to their cells of origin. Therefore, identifying and potentially reversing autologous diabetic EPC/EPC-EV dysfunction is of utmost importance before their clinical applications in ischemic tissue repair. Our data presented in the current study demonstrate that unlike non-diabetic EPC-derived EVs, EVs derived from diabetic EPCs lose the cardiac repair capabilities. We further show that part of this diabetic EPC-EV dysfunction is mediated by delivery of HDAC1 to recipient cells leading to deacetylation of Histone 3K9, an epigenetic mark associated with gene permissive chromatin state. Most, interestingly our studies suggest that inhibition of HDAC activity by HDAC inhibitor valproic acid, partly restores the functionality of both diabetic EPCs and their secreted EVs. The summary of the function and mechanisms of diabetic EPC-EV-induced reparative impairment in mice with cardiac injuries is shown in the graphical abstract.

As EVs reflect their parental stem cells' cargo and functional properties and since EVs are relatively stable, they appear attractive as cell-free therapeutics and conduits for gene and drug delivery for heart failure 38 In this perspective, EV therapy has been recently put forward as an alternative tool for cardiac repair and regeneration after MI 10, 11. However, despite these tremendous advantages, all EVs are not created equal, even when obtained from same kind of stem cells and complete functional characterization of EVs is needed before their potential clinical applications. Our previous studies revealed that EVs from EPCs obtained from the mouse model of chronic inflammation were functionally defective with altered EV protein cargo 13, 23. Information on the functional properties of EVs derived from diabetic cells on cardiac repair are scant, although few *in vitro* available studies have reported that EVs from diabetic source showed functional deficiencies largely by delivering specific microRNAs to recipient cells 39 Despite progress in discovering deficient EVs/ in response to stimuli, information remains limited on the effects of diabetes on EPC-derived EV function and its underlying mechanism.

Previously, we have reported that EPCs and other stem cells exposed to hyperglycemic culture conditions showed a dramatic alteration in EV RNA contents 14. In this study, we performed comprehensive studies demonstrating that diabetes impairs EPC-EV functions both *in vitro* cell culture system and *in vivo* in two models of myocardial injury. These results establish that healthy non-diabetic EPC-EVs substantially improve physiological and anatomical repair after MI; however diabetic EPC-EVs completely lose their acute cardioprotective and long-term cardiac reparative activities in the ischemic hearts. We also found that diabetic EPC-EVs augmented immune cell infiltration into the injured myocardium, likely due to the systemic and low level of inflammatory environment where EPCs may generate inflamed EVs in diabetic patients 40. It is important to note that only few studies investigated the protective effect of stem cell-derived EVs on acute myocardial protection after I/R injury. Our findings provided exciting evidence that healthy EPC-EVs provide a significant cardio-protection from acute I/R injury. These data implicate that the timely protective effect of EPC-EVs in the early stage of AMI may alleviate long-term cardiac maladaptive remodeling and improve cardiac function.

Epigenetic mechanisms such as DNA and histone modifications leading to altered gene expression are the critical regulatory pathways mediating the initiation, maintenance, and progression of both macro-and micro-vascular complications of diabetes and are also major contributors to epigenetic memory associated with diabetes 41-43. However, whether EVs derived from diabetic stem cells could potentially alter epigenetic mechanism-mediated gene regulation in recipient cells is not known. Therefore, in the current study, we aimed to investigate the epigenetic mechanism of diabetic EPC-EVs in the recipient MCECs. We observed that db/db EPC-EVs are enriched with HDAC1 which when delivered to recipient endothelial cells reduced transcription permissive H3K9Ac by increasing HDAC enzymatic activity. HDAC class I family is the established primary regulator for deacetylating H3K9 residue. Indeed, we found that diabetic-EPC-EVs both enhanced the HDAC enzymatic activity as well reduced H3K9 acetylation in recipient MCEC cells. Further, H3K9Ac ChIP sequencing experiments confirmed reduced H3K9ac mark and transcriptional repression of multiple transcripts involved in endothelial cell functions.

Preclinical effects of multiple HDAC inhibitors on MI and heart failure have been reported and showed their benefits on reducing the infarct area and improving LV function. Especially, Tian et al. showed that VPA administration protects heart function through Foxm1 pathway post-MI 34. Another study showed that VPA attenuates atrial remodeling and atrial fibrillation 44. In good accordance with previous studies, we observed that pre-treatment with VPA restored defective cardiac repair ability of diabetic EPCs and their EVs. Further studies utilizing HDAC1 KO mice with diabetes phenotype will be required to establish the conclusive role of HDAC1 is diabetes induced EPC dysfunctions. Nonetheless, our data suggest a critical role of post-translational histone acetylation changes mediated by diabetic EPC-EVs impair their functional and cardiac reparative activities. Our findings may have clinical implications since they suggest that inhibition of HDAC activity may partly reverse diabetes-induced EPC and EPC-EV dysfunctions and that pretreatment of diabetic stem cells with VPA prior to EV isolation may also enhance stem cell-EV-based therapeutic intervention. Alternatively, VPA therapy may be a helpful adjuvant when co-administered with diabetic stem cell/ EPC-EV. It is imperative to test the efficacy, timing, and dosing of VPA in future preclinical EPC-VPA combination treatment studies.

Finally, we would like to acknowledge the limitations of our current studies. Although we only examined H3K9Ac modification by diabetic-EPC-EVs, other components of EV cargo may also influence diabetic-EV-mediated recipient cell responses. EVs pack a multitude of bioactive molecules as their cargo including microRNAs and other noncoding RNAs, proteins, kinases, lipids, and metabolites. While establishing a functional role for each of the altered component of diabetic EPC-EVs is not feasible, a comprehensive analysis of diabetic EPC-EV cargo would be needed for identifying the pathways downstream of specifically altered biomolecules. Future studies will attempt at this endeavor. Despite these limitations, data presented in this study is provides a novel level of gene regulatory mechanism induced by diabetic EPC-EVs. Although we tested the efficacy of VPA-pretreatment on enhancing cardiac reparative activity of diabetic-EPCs, we did not test whether EVs from VPA-treated diabetic EPCs regain their cardiac reparative functions *in vivo* in MI models. Nonetheless, VPA-treated diabetic EPC-EV did regain their angiogenic and cell protective activities *in vitro*. In accordance with current notion that EVs secreted from stem cells largely mirror the functional properties of their parent cells 11-13, 15, and that adult stem/progenitor cells provide cardio-protection via secretion of EVs, our data with VPA-treated diabetic EPCs might reflect improved functionality of their secreted EVs. We believe that despite these limitations, our studies unravel a new mechanistic insight into Together, our data suggest that diabetes induced EPC and EPC-EV dysfunctions are in apert mediated by increased HADA1 activity and inhibition of HDAC by small molecule inhibitor partly restores the functions of diabetic EPC and their EV derivatives.

## Conclusions

In summary, we report that diabetes impairs the therapeutic capability of BM-EPCs as well as of their secreted EVs in two different models of cardiac injury. We demonstrate a new regulatory mechanism of HDAC-mediated epigenetic alteration by diabetic EPC-EVs that mediates functional repression in recipient cells. Inhibiting HDAC enzymes using the small molecule, VPA could rescue H3K9Ac level, which partly reverses cell/EV function. This study provides important implications that EPCs exhibit functionally impaired EVs in diabetes and, at least partly, through HDAC-mediated epigenetic mechanisms.

## Supplementary Material

Supplementary materials and methods, figures and tables.

## Abbreviations

AMI: acute myocardial infarction
CVD: cardiovascular disease
cTn-l: cardiac troponin I
CHIP-seq: chromatin immunoprecipitation sequencing
db/db: diabetic
db/+: nondiabetic
EC: endothelial cell
EV: extracellular vesicle
EPC: endothelial progenitor cell
EPC-EV: endothelial progenitor cell-derived extracellular vesicle
Echo: echocardiography
EF: ejection fraction
FS: fraction shortening
HSC: hematopoietic stem cell
HMVEC: human microvascular endothelial cell
H3K9Ac: histone H3 lysine 9 acetylation
H3K9Me3: histone H3 lysine 9 tri-methylation
HDACs: histone deacetylases
I/R: ischemia reperfusion
LAD: left anterior descending
LVEDV: left ventricle end-diastolic volume
LVESV: left ventricle end-systolic volume
MCEC: mouse cardiac endothelial cell
NRVM: neonatal rat cardiomyocyte
VPA: valproic acid
Sox12: SRY-box transcription factor 12
Pdgfd: Platelet-derived growth factor D
