# Supplementary material for: Diabetes impairs cardioprotective function of endothelial progenitor cell-derived extracellular vesicles via H3K9Ac inhibition
Source: Theranostics. 2022 May 21;12(9):4415–30. doi: 10.7150/thno.70821 (PMC9169353; doi:10.7150/thno.70821)
Supplement: Supplementary file 1 — Supplementary materials and methods, figures and tables. [file thnov12p4415s1.pdf]

ONLINE SUPPLEMENT

Diabetes impairs cardioprotective function of endothelial progenitor cell-derived extracellular vesicles via H3K9Ac inhibition

Grace Huang[1], Zhongjian Cheng[1], Alycia Hildebrand[1], Chunlin Wang[1], Maria Cimini[1], Rajika Roy[1], Anna Maria Lucchese[1], Cindy Benedict[1], Vandana Mallaredy[1], Ajit Magadum[1], Darukeshwara Joladarashi[1], Charan Thej[1], Carolina Gonzalez[1], May Trungcao[1], Venkata Naga Srikanth Garikipati[2,3], John W. Elrod[1,4], Walter J. Koch[1,4] and Raj Kishore[1,4]

Expanded Materials and Methods

Induction of myocardial infarction

The ligation of the left anterior descending (LAD) coronary artery was performed as a permeant MI model described previously [13]. Mice were under anesthesia using 2% isoflurane inhalation with an isoflurane delivery system (Viking Medical, Medford, NJ) during surgery. Immediately after LAD ligation, mice received an intramuscular injection of 1x10^9 db/+ EPC-EV particles (n = 10), 1x10^9 db/db EPC-EV particles (n = 10) or vehicle (n = 10) in a total volume of 20 μl at 3 different sites (basal anterior, mid anterior and apical anterior) in the peri-infarct area. Echocardiography for left ventricle (LV) functional studies before MI (baseline) and at 1-, 2-, 3- and 4-weeks after MI was recorded. Histology analyses for structural remodeling and capillary density were performed at 4 weeks post-MI.

Induction of ischemia reperfusion

Left coronary artery (LCA) ligation and ischemia reperfusion were performed as below. In brief, mice on C57/BL6N strain were anesthetized with 50 mg/kg of Ketamine combined with 8mg/kg of xylazine with 50U heparin and 200 μl of sterile saline. Once mice are fully

anesthetized, they are intubated and put on a ventilator. The heart was exposed via a left thoracotomy between the second and third ribs. A knot was placed around the LCA, and a PE10 tube that was 3-mm in length was placed on top of the LCA to allow for reperfusion. The heart was rinsed with sterile saline, and the wound was covered by parafilm for 45 minutes of the ischemic period. Immediately after the LCA was released (PE10 tube was released and the suture was removed), 5X10^9 db/+ EPC-EV, 5X10^^{9} diabetic EPC-EV, 2X10^^{5} nontreated or VPA-treated diabetic EPC-EV or saline in a total volume of 20 μl were given by intramyocardial injection. Mice were monitored during the whole surgery procedure. The ischemic zone was allowed to reperfuse for 24 hours. After LCA re-ligation, hearts were injected with 2% Evans blue dye to distinguish viable tissue from an area at risk to measure infarct size. 1-mm heart thick sections were cross-sectioned and incubated with 1% triphenyl tetrazolium chloride (TTC) (Sigma, T8877) for 5 min at 37°C to delineate infarcted tissue. All five 1-mm myocardial slices were weighed, imaged, and the second slices were fixed in 4% PFA. All values such as the infarct area, area at risk, and viable tissue were assessed using ImageJ as previously reported[23].

MCEC and Neonatal rat cardiomyocyte (NRVM) culture and treatment

MCEC was purchased from CEDARLANE (CLU510) and cultured on 0.2% Gelatin (Sigma)-coated dishes and maintained in 4.5 g/ml DMEM supplemented with 5% EV-depleted-FBS, Penicillin-Streptomycin (P/S), 10 mM HEPES (Sigma). For H_2O_2-induced cell apoptosis assay in endothelial cells, 1X10^^{4} MCECs were cultured in Exo-depleted culture media and treated with vehicle, 1X 10^6 db/+ EPC-EV, or 1X 10^^{6} diabetic EPC-EV for 24 hours following 100 μM H_2O_2 treatment for 4 to 5 hours. MCECs were then fixed in 4% paraformaldehyde (PFA) for TUNEL staining, and cell lysates were collected for Caspase 3/7 measurement (Promega, G811C). For VPA studies, MCECs were treated with vehicle, 1X 10^6 db/+ EPC-EV or 1X 10^6 diabetic EPC-EV or db/db EPC-EV from cells treated with 1 mM VPA for 24 hours. MCECs were lysed and subjected to Western blotting. NRVM was kindly provided by Dr. Walter J Koch's lab. For hypoxia and starvation study in expanding NRVMs, cells were isolated from one- to two-day-old rat pups and were cultured in Ham's F-10 media (Corning) supplemented with 10% horse serum, 5% FBS. After 24 hours of isolation, media was changed to F-10 media supplemented with 5% Exo-depleted FBS. After NRVM was attached completely and presented beating phenotype, 1X10^6 NRVM were treated with vehicle, 1X 10^8 db/+ EPC-EV, or 1X 10^8 diabetic EPC-EV for 24 hours in serum-free F-10 media following incubation in the hypoxia chamber for 48 hours. NRVM were then fixed in 4% PFA for

TUNEL staining, and cell lysates were used for Caspase 3/7 measurement (Promega, G811C).

Chromatin immunoprecipitation sequencing (ChIP-seq)

MCECs were fixed with 1% formaldehyde for 15 min and quenched with 0.125 M glycine after db/+ or db/db EPC-EV treatment for 24 hours and then subjected to ChIP-seq (Active Motif, Inc.) In brief, chromatin was isolated by adding lysis buffer, followed by disruption with a Dounce homogenizer. Lysates were sonicated, and the DNA sheared to an average length of 300-500 bp with Active Motif's EpiShear probe sonicator (53051) and cooled sonication platform (53080). Genomic DNA (Input) was prepared by treating aliquots of chromatin with RNase, proteinase K, and heat for de-crosslinking, followed by SPRI beads clean up. Clariostar quantified eluted DNA. Extrapolation to the original chromatin volume allowed quantitation of the total chromatin yield. An aliquot of chromatin (30 mg) was precleared with protein A agarose beads (Invitrogen). Genomic DNA regions of interest were isolated using 5 ul antibody against H3K9Ac (Active Motif cat# 39917). Complexes were washed, eluted from the beads with SDS buffer, and subjected to RNase and proteinase K treatment. Crosslinks were reversed by incubation overnight at 65°C, and ChIP DNA was purified by phenol-chloroform extraction and ethanol precipitation.

Illumina sequencing libraries were prepared from the ChIP and Input DNAs using the standard consecutive enzymatic steps of end-polishing, dA-addition, and adaptor ligation using Active Motif's custom liquid handling robotics pipeline. After the final 18 cycle PCR amplification step, the resulting DNA libraries were quantified and sequenced on Illumina NexSeq 500. Sequences (75 bp, single-end) were aligned to the mouse genome (mm10) using the BWA algorithm (default settings). Duplicate reads were removed, and only uniquely mapped reads (mapping quality ≥ 25) were used for further analysis. Alignments were extended in silico at their 3'-ends to a length of 200 bp, which is the average genomic fragment length in the size-selected library, and assigned to 32-nt bins along the genome. The resulting histograms (genomic "signal maps") were stored in BigWig files. Peaks were identified using the MACS 2.1.0 algorithm at a cutoff of p-value 1e-7, without control file, and with the –nomodel option. Peaks that were on the ENCODE blacklist of known false ChIP-Seq peaks were removed. Signal maps and peak locations were used as input data to Active Motifs proprietary analysis program, which creates Excel tables containing detailed information on sample comparison, peak metrics, peak locations, and gene annotations.

SUPPLEMENTARY FIGURES

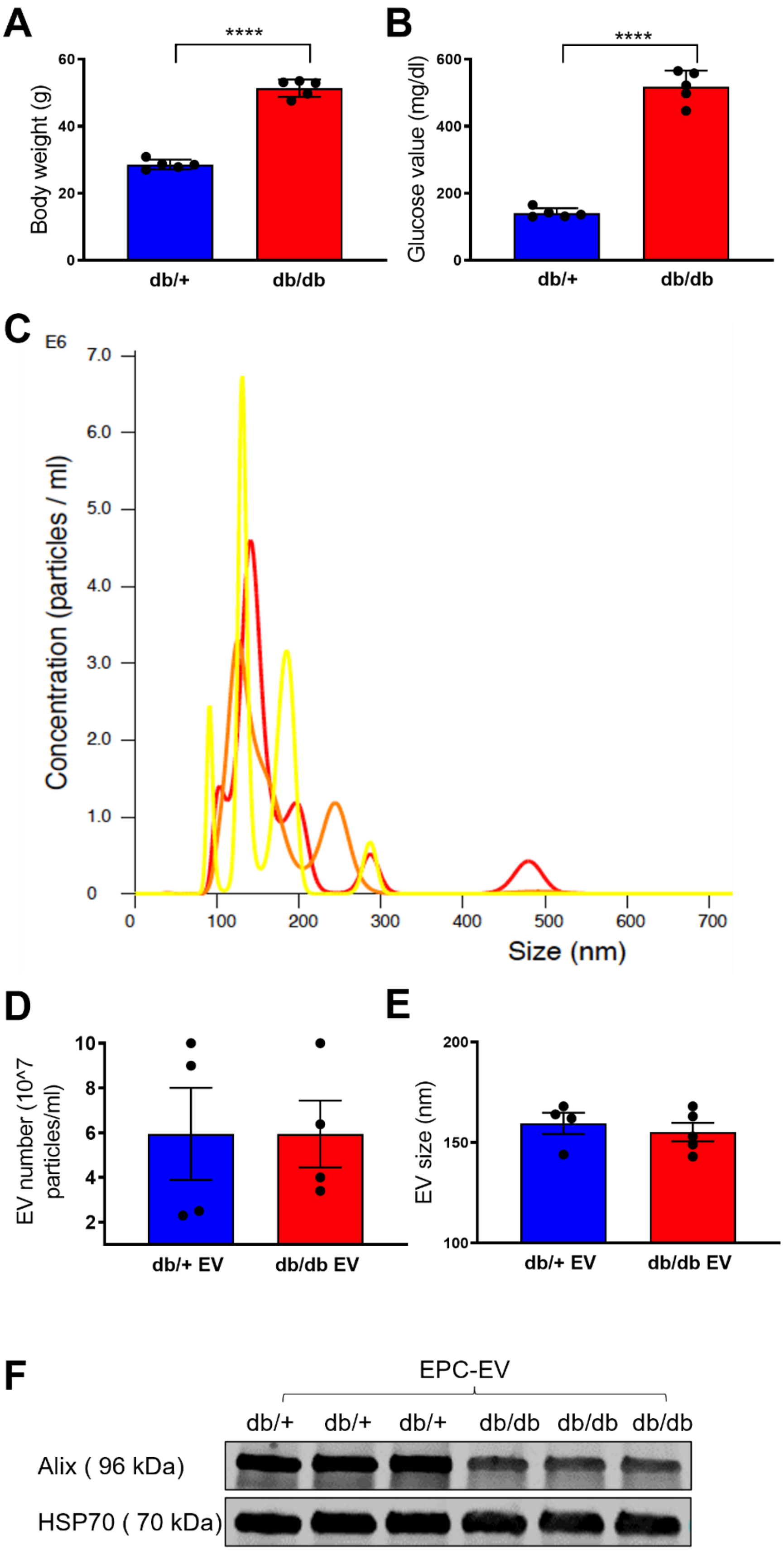

A
B
C
D
E
F
Body weight (g)
Glucose value (mg/dl)
****
db/+
db/db
E6
Concentration (particles / ml)
Size (nm)
EV number (10^7 particles/ml)
EV size (nm)
db/+ EV
db/db EV
EPC-EV
Alix (96 kDa)
HSP70 (70 kDa)

Figure S1: Physiological parameters and EPC-EVs characterization from diabetic and non-diabetic mice. (A) The bodyweight of 10-week-old mice was measured before EPC isolation. (B) Whole blood was collected without pre-fasting for blood glucose measurement using the OneTouch ULTRA2 glucose meter. (C-D) EV particle number of db/+ and db/db EPCs is in average of 5X10^7 from 3X10^5 number of cells and (C-E) with a diameter of approximately 150 nm measured by Nanosight. (F). Identification of EVs by EV marker proteins Alix and HSP70. Both db/+ and db/db EPC-EVs expressed Alix and HSP70. All Data shown as mean ± SEM. n > 3. **** P<0.0001.

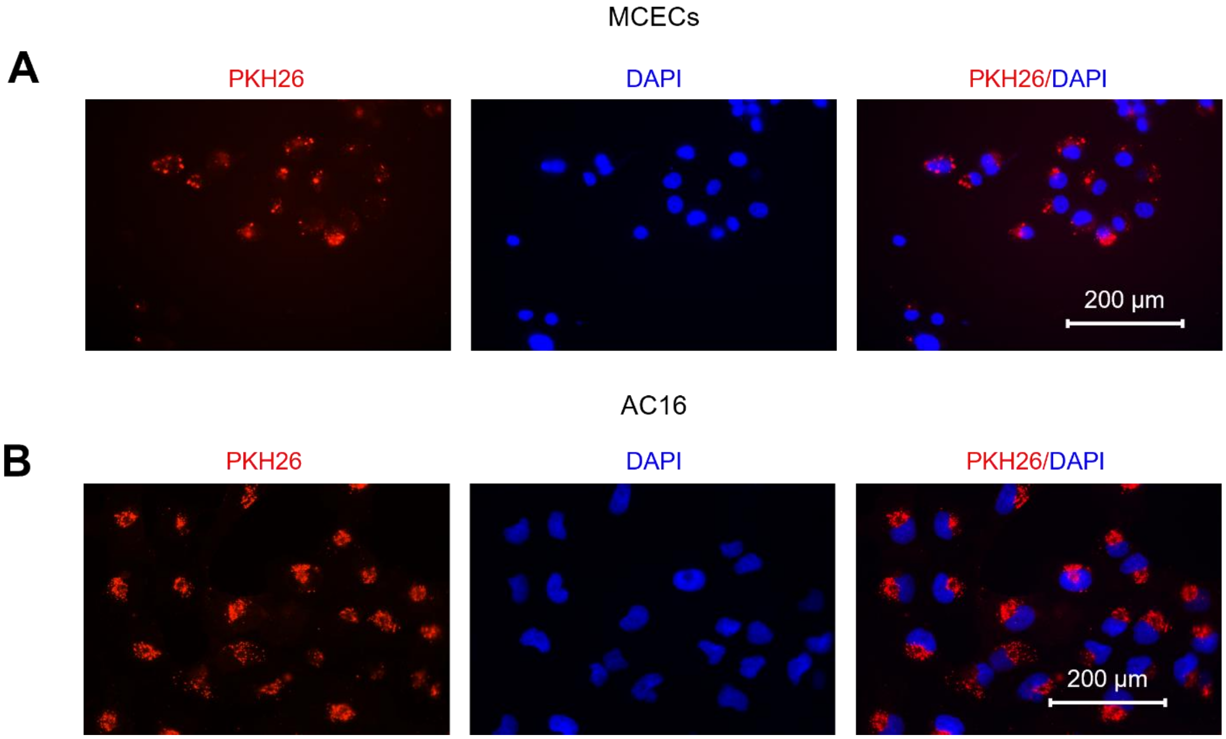

Figure S2: Uptake of PKH26-labled EPC-EVs by MCECs (A) and AC16 cells (B). EPC-EVs from db/+ mice were resuspended in 1 ml Diluent C and mixed with 1 ml stain solution (1 ml Diluent C + 4 µL PKH26) and incubated for 4 min on ice. Then equal volume of 1% BSA was added to stop the labeling reaction. Then the EVs were diluted with 1xPBS andcollected on a 30% sucrose-D2O solution with ultracentrifugation (100,000g for 1 hour). Following separation on the sucrose gradient, the EVs were washed in 1xPBS and the pelleted EVs were suspended in desired volume of PBS and added to MCECs (A) and AC16 cardiomyocytes (B) cells. After 1.5 hrs, the EV-treated MCECs and AC16 cells

were washed with 1xPBS and fixed with 4% PFA for 10 min on ice. Images were acquired using the Niko Eclipse Ti Florescence microscope using 20x objectives.

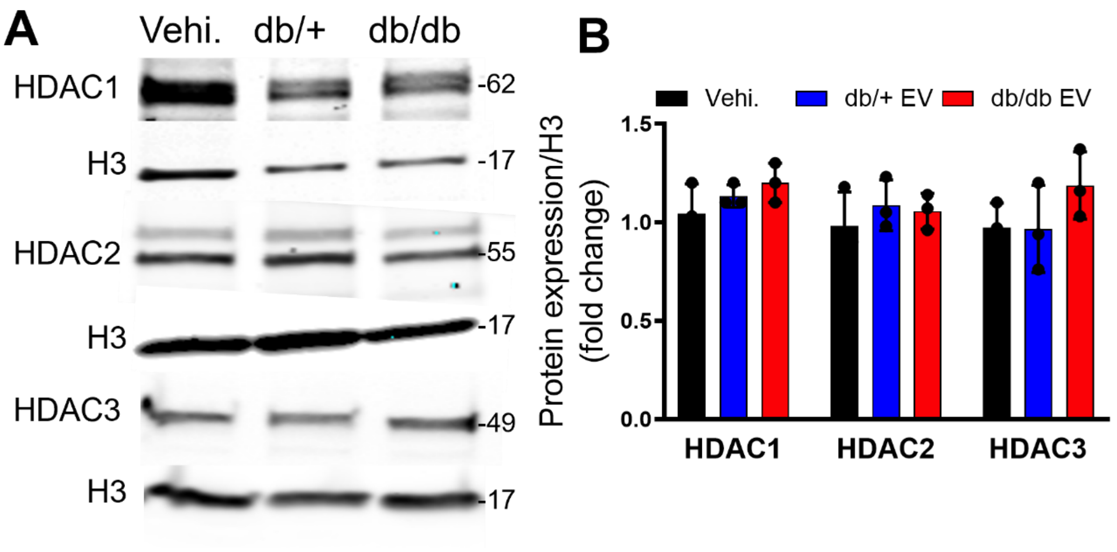

Figure S3. Non-Diabetic EPC-EVs and diabetic EPC-EVs do not not significantly change HDAC1, HDAC2 and HDAC3 expression in recipient MCECs. (A) MCECs were treated with vehicle, db/+ or db/db EPC-EVs for 24 hours. Cells were collected, lysed, and subjected to Western blotting using HDAC1, HDAC2, and HDAC3 antibodies. (B) Quantification analysis showed that db/db-EPC-EV trend to increase HDAC1-3 protein but did not achieve statistical significance in protein expression. All Data are shown as mean ± SEM. n=3 for each group. HDAC, histone deacetylases. Vehi., vehicle.

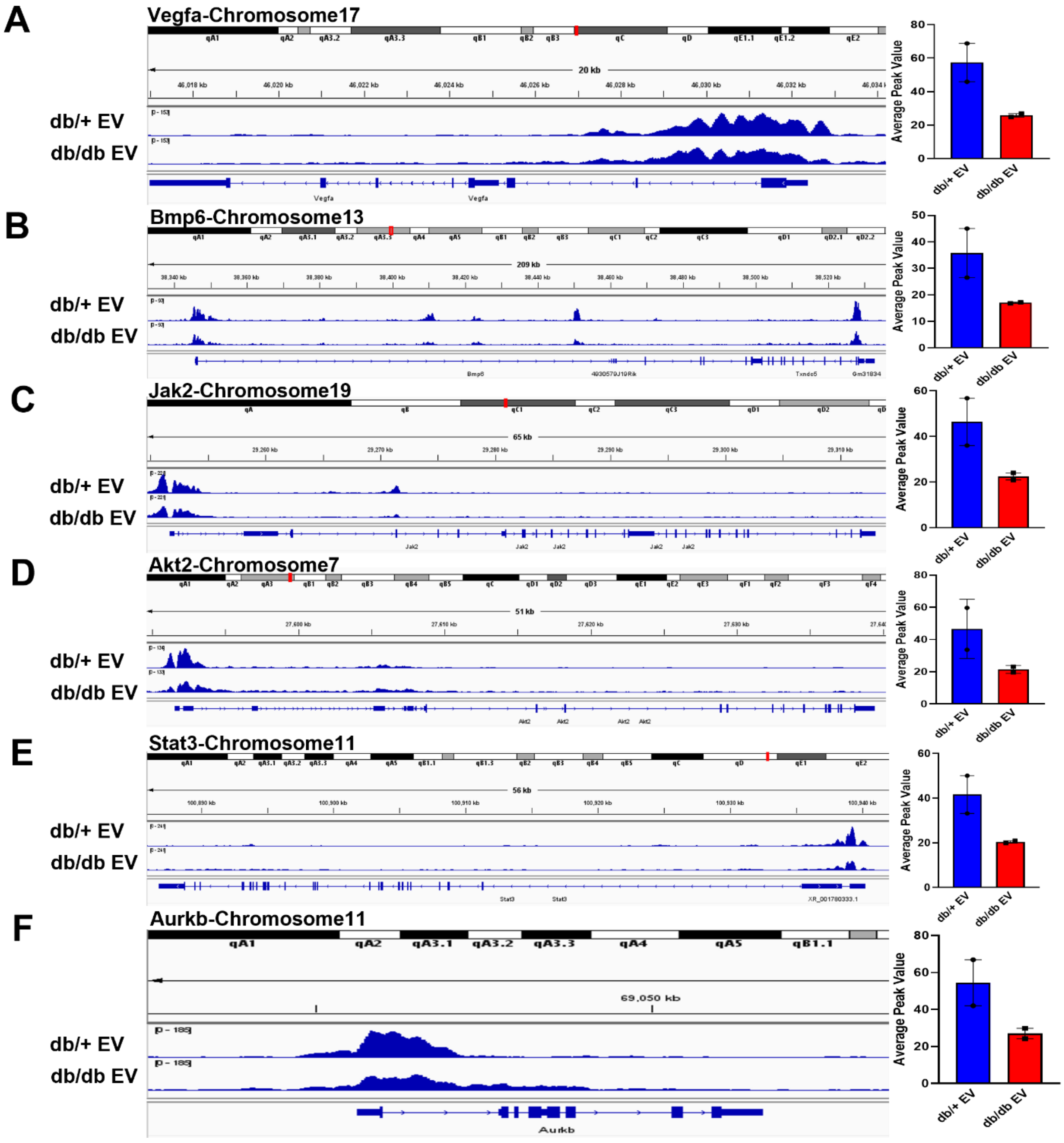
A
Vegfa-Chromosome17
20 kb
db/+ EV
db/db EV
Vegfa
Average Peak Value
B
Bmp6-Chromosome13
209 kb
db/+ EV
db/db EV
Average Peak Value
C
Jak2-Chromosome19
65 kb
db/+ EV
db/db EV
Average Peak Value
D
Akt2-Chromosome7
51 kb
db/+ EV
db/db EV
Average Peak Value
E
Stat3-Chromosome11
56 kb
db/+ EV
db/db EV
Average Peak Value
F
Aurkb-Chromosome11
69,050 kb
db/+ EV
db/db EV
Aurkb
Average Peak Value

Figure S4. db/db EPC-EV decrease H3K9Ac level at TSS of survival/proliferative gene in MCECs. (A-F) Integrative genomics viewer (IGV) software was used to view the fragment density of H3K9Ac (y-axis) aligned along with the gene coordinates (x-axis). Data are presented as bar graphs representing the mean. n=2 for each group. Vehi., vehicle.

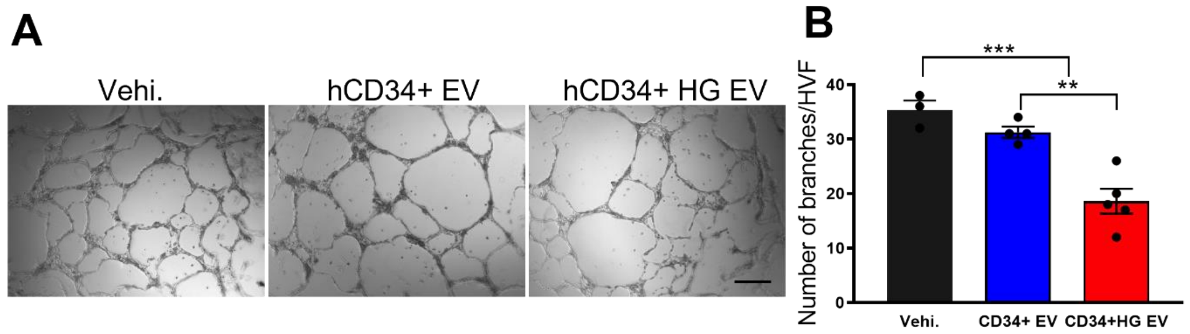

Figure S5. Hyperglycemia alters human hematopoetic stem cell-derived EV function. (A) Human CD34+ cell-EVs under hyperglycemic insult reduced tubulogenesis in HMVECs. Tubulogenesis in human microvascular endothelial cells treated with vehicle, CD34+ cell-EV or CD34+ cell HG-EV. (B) Quantification analysis of branch number per HVF. Scale bar = 100 mm. All Data are shown as mean ± SEM, n≥3 for each group. *** *P*<0.001, ***P*<0.01. hHSC, human hematopoietic; stem cell; HG, high glucose; HVF, high visual field; Vehi., vehicle.

SUPPLEMENTAL TABLES

RT-PCR primer (mRNA)		
**Primer name**	**Primer sequence forward**	**Primer sequence reverse**
Sox12	GGAGACGGTGGTATCTGGG	ATCATCTCGGTAACCTCGGGG
Pdgfd	TACAGTTGCACTCCCAGGAAT	CTTCCAGTTGACAGTTCCGCA
Gapdh	CGTGTTCCTACCCCCAATGT	TGTCATCATACTTGGCAGGTTTCT

Table S1. Primer sequences. Sequences used for RT-PCR in mouse samples.

Antibody	Company	Product Number	Species	Primary Ab Conc.	Secondary Ab Conc.
H3	Cell Signaling	14268S	Mouse	1:2,000	1:10,000
H3K9Ac	Active Motif	#39137	Rabbit	1:1,000	1:10,000
H3K9Me3	Cell Signaling	#13969	Rabbit	1:500	1:5,000
HDAC1	Cell Signaling	34589T	Rabbit	1:1,000	1:10,000
HDAC2	Cell Signaling	57156T	Rabbit	1:1,000	1:10,000
HDAC3	Cell Signaling	85057T	Rabbit	1:1,000	1:10,000
Actin	Cell Signaling	#4970L	Rabbit	1:2,000	1:10,000
Donkey anti-Rabbit	Li-Cor	926-32213			1:10,000
Donkey anti-Mouse	Li-Cor	926-68072			1:10,000
Alix	Proteintech	12422-1-AP	Rabbit	1:2000	1:10,000
HSP70 (3A3)	Santa Cruz Biotechnology	Sc-32239	Mouse	1:500	1:10,000

Table S2. Antibody information. Antibody information for Western Blot analysis.

		Vehicle		Db/+ EPC-EV		Db/db EPC-EV	
		Mean	SEM	Mean	SEM	Mean	SEM
Baseline	Heart Rate (bpm)	483.39	20.70	455.93	13.52	463.61	24.52
	ESD (mm)	2.33	0.06	2.65	0.08	2.61	0.11
	EDD (mm)	3.99	0.12	4.10	0.06	4.11	0.11
	ESV (µl)	18.88	1.27	26.36	1.93	25.59	2.20
	EDV (µl)	70.05	4.88	74.38	2.69	75.44	4.37
	SV (µl)	51.18	4.06	48.02	2.24	49.84	2.80
	EF (%)	72.79	1.40	64.15	2.31	66.09	2.13
	FS (%)	41.42	1.26	34.91	1.65	36.32	1.65
	CO (ml/min)	24.4	1.40	21.76	1.06	22.85	1.29
1 Week	Heart Rate (bpm)	460.34	24.11	516.75	14.17	520.74	16.48
	ESD (mm)	4.01	0.13	3.91	0.19	3.83	0.17
	EDD (mm)	4.90	0.09	4.71	0.15	4.64	0.17
	ESV (µl)	70.92	5.39	69.15	7.38	65.37	6.50
	EDV (µl)	113.51	4.96	104.53	7.40	101.20	8.05
	SV (µl)	42.59	2.52	35.39	2.32	35.82	2.67
	EF (%)	37.93	2.68	35.43	3.47	36.23	3.06
	FS (%)	18.44	1.51	17.22	1.89	17.55	1.65
	CO (ml/min)	19.67	1.72	18.20	1.15	18.52	1.37
2 Weeks	Heart Rate (bpm)	493.92	26.06	519.20	15.34	535.41	30.94
	ESD (mm)	4.25	0.19	4.04	0.18	4.50	0.29
	EDD (mm)	5.11	0.13	5.04	0.19	5.34	0.24
	ESV (µl)	82.18	7.90	74.40	7.79	97.83	13.36
	EDV (µl)	124.97	7.31	123.46	10.29	141.71	14.23
	SV (µl)	42.80	2.73	49.06	3.47	43.88	3.32
	EF (%)	34.85	3.54	40.47	2.02	33.59	3.92
	FS (%)	16.91	1.97	19.92	1.13	16.40	2.15
	CO (ml/min)	20.92	1.30	25.22	1.70	23.62	2.39
3 Weeks	Heart Rate (bpm)	470.48	25.99	511.27	16.02	496.30	16.11
	ESD (mm)	4.37	0.28	3.84	0.20	4.86	0.20
	EDD (mm)	5.22	0.22	4.93	0.19	5.51	0.20
	ESV (µl)	89.49	14.16	67.05	8.26	113.51	11.06
	EDV (µl)	132.56	13.24	117.17	10.34	150.69	12.74
	SV (µl)	43.07	4.89	50.12	3.09	37.18	3.84
	EF (%)	34.29	4.49	44.63	2.60	24.54	2.06
	FS (%)	16.72	2.47	22.38	1.50	11.52	1.05
	CO (ml/min)	20.12	2.47	25.27	1.30	18.28	1.77

4 Weeks	**Heart Rate (bpm)**	413.49	26.97	473.25	16.10	485.46	17.82
	**ESD (mm)**	4.27	0.22	4.29	0.22	5.05	0.22
	**EDD (mm)**	4.94	0.21	5.38	0.18	5.66	0.19
	**ESV (µl)**	83.86	10.31	86.50	10.43	124.41	12.96
	**EDV (µl)**	116.78	11.34	143.22	11.36	160.15	12.87
	**SV (µl)**	32.91	5.72	56.72	3.34	35.74	1.70
	**EF (%)**	28.73	4.14	41.30	3.03	23.43	1.99
	**FS (%)**	13.68	2.14	20.66	1.71	10.96	0.96
	**CO (ml/min)**	13.37	2.11	26.94	1.99	17.26	0.89

Table S3: Echo data table for vehicle, db/+ EPC-EV and db/db EPC-EV injected mice following MI surgery – Mean and standard error of the mean for variables measured using VevoStrain analysis at baseline, 1-, 2-, 3- and 4- weeks post-surgery. n=7 for vehicle group, n=13 for db/+ EPC-EV group, n=11 for db/db EPC-EV group.

Gene List	db+ EPC-1	db+ EPC-2	dbdb EPC-1	dbdb EPC-2	pvalue
1110034G24Rik	37.425	50.475	21.350	22.225	0.00169
1700017L05Rik	43.933	51.533	18.267	22.667	0.02251
1700023L04Rik	26.880	40.360	17.920	15.120	0.01935
1700063D05Rik	44.583	62.083	29.750	23.167	0.04410
2310008H04Rik	36.812	49.938	19.000	18.188	0.02687
2310008H04Rik	26.809	36.095	13.809	15.524	0.00969
2310022A10Rik, Akt2	33.611	59.611	19.722	23.111	0.00797
2310043O21Rik	33.647	50.294	21.941	19.059	0.09055
2310047M10Rik, Tmem107	43.182	76.636	13.818	24.182	0.00448
2810055G20Rik	31.656	53.875	18.188	16.312	0.00055
3830408C21Rik	32.683	50.342	18.000	20.146	0.00002
4930525G20Rik, Zfp273	43.621	62.345	27.000	25.793	0.00027
4930573C15Rik	51.625	52.688	24.125	20.250	0.01100
4933405D12Rik, 1810037I17Rik, Usp53	44.750	68.750	26.583	28.583	0.03842
4933433H22Rik	58.000	70.538	28.000	30.000	0.01639
5033411D12Rik, Mplkip	30.120	44.160	18.000	17.720	0.06372
5830418K08Rik	46.733	74.200	31.600	23.867	0.01297
6030466F02Rik	26.435	43.522	16.956	14.696	0.00788
8030442B05Rik, Gm13293	43.500	58.182	19.727	25.409	0.00340
9130015A21Rik	25.242	34.667	11.788	11.424	0.00046
9230104L09Rik	42.429	63.184	25.020	23.694	0.00000
9330175M20Rik	32.467	49.133	13.467	14.733	0.00047
9330175M20Rik	45.522	56.304	20.478	25.391	0.00324
A330021E22Rik	21.350	39.800	13.250	10.950	0.02051
A330021E22Rik	33.056	42.611	15.833	16.722	0.01721
Abca1	38.857	45.943	22.714	18.771	0.00197
Abca12	37.294	61.647	20.941	20.118	0.01696
Abca4	43.080	56.800	21.040	27.480	0.00511
Abcd4	45.368	59.579	26.053	23.000	0.09566
Ablim2, Afap1	44.083	76.917	23.000	24.917	0.00951
Acap2	32.214	60.071	22.214	22.643	0.25609
Acsl4	26.783	36.696	13.652	14.261	0.01544
Actn1	31.950	48.750	19.150	18.550	0.02314
Actr10, Psma3	51.136	73.818	26.409	29.818	0.00006
Actr1b, 4933424G06Rik	25.571	57.714	14.571	19.143	0.02029
Actrt3, Mynn	38.562	53.188	22.500	17.938	0.01367
Adamtsl3	24.345	32.414	13.483	14.414	0.09991
Ahnak	46.400	49.200	29.867	17.333	0.09005
AK157302, Zkscan8	38.393	57.036	17.464	20.929	0.00020
Ak5	22.000	39.692	14.846	14.346	0.01503
Akap6	29.125	39.562	16.031	14.594	0.00067

Akirin2	28.278	46.111	20.944	15.944	0.15544
Akr1b3	51.524	64.952	30.905	26.524	0.00290
Anapc1	37.000	45.737	18.526	21.632	0.04812
Anapc1, Mertk	37.387	57.452	24.387	22.355	0.00100
Anxa1	44.260	62.040	22.700	27.180	0.00000
Anxa3	53.200	71.067	30.067	23.533	0.01218
Ap4e1	36.885	54.308	17.808	20.615	0.00097
Apbb2	30.226	39.323	18.323	16.194	0.01975
Apoa5, Zfp259, Bud13	42.077	64.231	21.769	24.308	0.02802
Appl2	24.980	41.020	18.061	14.531	0.00277
Arhgap26	23.333	33.208	14.833	13.375	0.04539
Arhgef28	26.448	31.414	14.414	14.448	0.02534
Arid5b	21.891	36.239	13.000	14.522	0.00499
Arl14epl	33.741	50.407	19.444	17.000	0.00173
Armc2	25.857	52.571	15.071	19.143	0.04696
Armc2	27.222	41.926	14.074	16.259	0.00700
Armc9	36.333	47.714	18.238	19.809	0.00693
Arnt2	37.750	50.188	18.250	22.062	0.01538
Atad2, Wdyhv1	39.062	60.500	22.000	20.875	0.01551
Atf7ip	26.615	31.654	12.039	13.423	0.00638
Atg16l1	30.625	46.250	19.875	15.375	0.07670
Atp5g1	40.333	60.067	25.000	25.133	0.05209
Atp5g2, Mir688	27.000	44.056	15.111	17.833	0.02282
Atp6v0a4	41.857	61.429	19.786	24.000	0.00949
B3gntl1	45.700	67.850	21.650	25.400	0.00186
Bbs4	45.118	64.706	29.765	23.235	0.01184
BC053749, Hspb6, Lin37, Psenen	48.931	68.517	29.000	29.310	0.00062
Bdh1	34.000	49.357	16.571	21.571	0.04776
Bicc1	24.763	37.500	14.658	15.921	0.00356
Blm	50.000	83.056	34.556	31.500	0.00145
Bmp6	26.571	45.082	16.857	17.265	0.00073
Bmper	27.542	49.750	18.083	20.375	0.05625
Bnc2	31.792	55.167	17.625	22.792	0.00841
Brca1, Nbr1	94.000	137.947	52.842	53.842	0.00002
Btd	41.833	58.833	21.250	21.083	0.00925
Btg1	20.689	28.844	12.178	11.111	0.00424
C1rl	23.706	46.706	14.882	18.765	0.02085
Cacul1	64.188	109.188	40.062	41.500	0.00441
Capn7	35.750	62.938	24.188	23.688	0.12398
Casp1, Casp4	23.538	33.692	15.692	12.500	0.04544
Casp8ap2	41.077	79.231	30.923	28.462	0.04477
Ccdc134, Srebf2	35.615	59.154	19.154	24.462	0.02225
Ccdc63	55.706	83.294	34.294	34.441	0.00005

Ccnyl1	42.500	55.071	23.214	24.143	0.07629
Cdh23, 4632428N05Rik	28.556	43.833	18.000	14.722	0.02043
Cenpp, Ogn	24.167	30.083	13.667	11.000	0.04519
Cenpp, Omd	22.972	36.278	12.278	13.972	0.00360
Cep112	38.667	50.467	19.533	22.333	0.09686
Cep128	40.583	59.167	27.083	22.000	0.07933
Cerk	28.133	46.667	16.800	20.467	0.13411
Chn2	38.312	48.562	19.812	19.875	0.01399
Chn2	50.818	69.409	27.546	31.046	0.00324
Chp1, 1700020I14Rik	49.882	63.941	27.471	23.882	0.01494
Cln5	35.684	55.053	20.158	19.526	0.00440
Col18a1	36.000	52.158	20.000	20.000	0.02438
Col1a2	50.733	70.600	26.733	26.200	0.00270
Col3a1	45.308	59.615	21.000	21.462	0.00613
Col3a1	48.015	70.076	28.758	30.015	0.00000
Col5a2	24.790	38.526	15.368	12.053	0.02119
Cox18, Ankrd17	52.833	73.583	22.583	33.250	0.01777
Cpe	33.895	46.316	20.053	19.526	0.01227
Creb3l2	46.684	82.053	31.895	32.105	0.00821
Cstl1	36.692	66.077	24.385	21.615	0.01275
Ctc1, Aurkb	42.000	66.909	24.227	29.864	0.00296
Ctif	55.455	76.454	37.182	26.454	0.11250
Cttnbp2nl, 4930564D02Rik	37.200	67.133	21.933	26.200	0.02840
Dact3, Gng8	48.769	57.692	21.615	24.923	0.03050
Ddc, 1700042O10Rik	33.692	59.538	16.769	27.077	0.07058
Ddx4, Slc38a9	54.294	75.588	31.059	33.176	0.02334
Ddx52, Synrg	53.933	74.133	34.867	27.067	0.02392
Derl1	24.000	38.870	15.087	13.913	0.00549
Dhrs3	26.462	46.769	19.205	15.026	0.00060
Dhx33, Derl2	34.368	48.000	21.474	18.895	0.02224
Disp1	26.083	37.542	12.625	12.125	0.00364
Dnajc16, Casp9	37.357	49.500	20.786	20.929	0.08795
Dock5	38.471	50.059	20.412	22.235	0.06985
Dpep2	27.471	42.059	15.706	18.588	0.03633
Dtx2, Upk3b	48.077	68.538	23.308	21.692	0.00330
E030003E18Rik	43.714	72.643	29.929	24.071	0.00953
Ebpl, Kpna3	35.619	54.857	22.095	21.191	0.03464
Eef2k, Polr3e	25.250	35.100	14.550	13.800	0.06012
Elf1	19.633	26.400	10.067	12.567	0.04349
Epha7	38.222	52.481	21.852	22.185	0.00267
Esyt1, Zc3h10	33.000	54.700	21.850	18.950	0.00750
Eya4	28.682	39.318	16.864	12.500	0.00823
Eya4	25.310	35.448	14.621	13.931	0.02410

Fam107b	57.850	88.350	39.900	32.900	0.00133
Fam122b, Fam122c	33.579	53.789	22.579	19.474	0.01501
Fam129a	17.750	31.375	8.458	8.083	0.00049
Fam13c	29.529	50.647	17.000	18.882	0.02464
Fam168a	38.667	44.333	15.833	21.389	0.01133
Fam169b	36.448	45.069	18.517	15.448	0.00494
Fam203a, Tssk5, Mroh1	48.600	63.267	29.600	23.533	0.03501
Fam35a, Glud1	40.640	68.360	24.280	30.200	0.01751
Fam71f1	20.147	30.147	11.618	11.794	0.01006
Fbxo47	35.045	42.409	20.500	15.364	0.00684
Fbxo5, Mtrf1l	57.917	72.750	33.000	26.917	0.01250
Fcgr4	52.938	76.625	32.500	30.562	0.01095
Fez2	26.973	37.973	14.757	17.676	0.03048
Fhl3, Sf3a3	49.000	60.923	25.538	26.000	0.00431
Fhl3, Sf3a3	38.217	63.304	24.174	26.000	0.00545
Fiz1, Zfp524, Zfp865	51.778	80.611	32.667	32.500	0.01112
Fndc7	18.097	34.839	11.290	11.613	0.01249
Fnip1	25.000	47.353	17.235	17.529	0.09493
Fras1	36.857	44.619	15.809	21.095	0.01140
Frmd4b	27.500	49.857	17.857	18.286	0.05070
Frmd4b	28.304	34.304	15.435	15.609	0.08462
Ftx	32.696	46.413	20.217	18.087	0.00009
Gm11351	18.226	29.645	12.839	11.000	0.01769
Gm12216	35.222	52.500	23.833	18.556	0.03137
Gm19651	32.062	56.625	20.562	23.688	0.01244
Gm19705, Gm16880	37.913	55.217	22.261	20.696	0.00135
Gm20139	31.212	48.424	16.697	15.242	0.00009
Gm21552	38.357	57.857	26.000	20.214	0.01366
Gm4613	32.357	46.464	19.393	18.607	0.00308
Gm5082	28.059	34.324	17.147	12.471	0.01908
Gm5084	33.875	44.500	18.688	19.688	0.02784
Gm5503	37.545	53.727	21.546	16.591	0.00086
Gm5546	28.346	39.923	14.808	16.846	0.01307
Gm5627	42.176	56.529	18.706	20.177	0.00791
Gm6116	33.000	49.471	20.353	20.118	0.05362
Gm6185	37.312	56.000	23.750	22.875	0.03020
Gm7550	28.920	36.400	11.880	15.040	0.00223
Gm7550	45.474	67.526	24.842	22.368	0.00044
Gm8883	33.250	57.667	21.000	17.250	0.01667
Gm9895	27.412	46.059	16.059	18.294	0.07456
Gnal, Chmp1b, Mppe1	23.192	28.231	13.308	12.077	0.11728
Gnl3l	38.897	51.552	17.621	21.276	0.00171
Gnl3l	29.000	42.767	18.567	17.233	0.01158

Gnpda1, Gm4949	33.870	42.778	17.259	19.315	0.00001
Golm1	38.450	47.800	20.300	14.650	0.00177
Gpm6a	31.300	47.600	15.167	23.333	0.02282
Gpr155	47.167	68.111	30.222	26.111	0.00754
Grin3b, Tmem259, Cnn2	55.421	95.790	30.368	35.632	0.00484
Grk6	36.423	45.615	17.923	19.923	0.00375
Gsg1	20.292	37.167	12.917	12.917	0.00590
Gulp1	28.191	44.143	16.286	15.952	0.00805
Gzmm, Bsg	46.806	65.000	27.968	27.194	0.00070
Hbegf, Slc4a9	39.375	62.938	24.000	24.812	0.04544
Hdac1, Lck	49.308	61.615	29.385	22.462	0.02171
Hdac7	49.200	60.867	30.867	23.867	0.03770
Hdgf, Mrpl24	31.885	54.077	19.808	20.346	0.01896
Hibch	33.667	50.800	23.333	17.800	0.04971
Hist1h3c, Hist1h2bb, Hist1h2ab, Hist1h3b, Hist1h4b, Hist1h4a, Hist1h3a	31.087	35.739	16.217	16.478	0.04498
Hmgn5	18.759	30.552	10.517	12.621	0.01543
Idh1, Pikfyve	59.556	87.926	35.185	36.111	0.00005
Ifnar2	37.618	54.118	24.618	21.088	0.00304
Ikzf2	42.151	62.758	29.788	21.606	0.00052
Incenp	23.286	35.191	15.381	12.286	0.02498
Ing3	32.250	49.050	18.500	18.450	0.00699
Ints10	43.947	68.632	25.632	29.053	0.00996
Irf2bpl	44.750	70.167	24.500	28.583	0.02591
Itpr1	23.818	33.773	13.500	12.727	0.02871
Ivd	37.526	55.158	16.210	22.632	0.00304
Jak2	36.045	56.773	24.046	20.909	0.01551
Kcnab1	27.750	44.438	13.500	13.312	0.02480
Kcnab1	23.394	33.576	12.273	14.546	0.00395
Kcnab1	41.138	60.207	24.207	26.241	0.00599
Kctd17, Tmprss6	45.500	74.000	26.375	30.188	0.00550
Kif13b	23.600	27.500	12.667	12.667	0.01493
Kif2c	41.387	65.452	26.710	25.839	0.00089
Klf6	24.700	37.433	14.367	15.100	0.03453
Kpna1, Wdr5b, Fam162a	31.333	50.267	21.667	15.800	0.06452
Lamc2	24.667	35.433	15.767	13.700	0.02055
Lims1	34.786	53.143	21.821	18.429	0.00087
Lims1	22.273	36.500	12.273	16.318	0.04796
Lnpep	30.839	46.387	16.129	21.613	0.00218
LOC100861595	29.485	41.455	15.879	17.394	0.01381
Lrif1	27.833	42.833	15.944	16.667	0.03171
Lrp2	24.500	41.944	17.500	14.722	0.04275
Lrrk1	25.500	42.917	16.792	16.958	0.02018

Lrrn4	55.368	79.947	22.579	26.632	0.00001
Lrrn4	25.400	43.033	13.433	18.000	0.00874
Lsm11	31.357	54.214	21.143	19.429	0.04722
Ltn1	25.200	39.850	14.400	17.200	0.03821
Lyst	32.312	48.188	13.188	12.375	0.00020
Mad2l1	51.312	64.500	29.812	24.188	0.01465
Maml3	40.882	57.176	24.412	24.471	0.00133
March3	48.214	71.000	25.429	31.286	0.01704
Mast4	35.050	43.850	17.800	20.850	0.03382
Mdfic	22.688	45.625	14.125	18.312	0.14467
Mertk	46.794	61.324	24.588	29.323	0.00421
Metap1d	23.312	44.375	14.562	15.625	0.06416
Mettl20	23.381	34.476	11.191	15.191	0.03759
Mir5118	45.581	59.065	26.936	24.355	0.00016
Mog	45.769	65.333	28.282	26.692	0.00002
Morf4l2, BC065397	43.171	58.657	26.857	21.543	0.00025
Mpp7	31.444	47.833	15.333	17.944	0.01757
Mrpl45	50.480	76.800	30.400	31.920	0.00063
Msantd4	54.667	77.571	36.095	29.667	0.00648
Mtcp1, Brcc3	33.323	38.581	16.871	19.064	0.02118
Myl7, Gck	34.238	47.429	19.191	20.143	0.03360
Myo1e	24.480	34.840	12.680	14.920	0.01225
Myo5a	21.714	39.333	16.809	11.619	0.03491
Mzf1	45.111	81.889	33.556	27.111	0.09962
N4bp2l2, Pds5b	50.143	71.071	27.500	26.786	0.00474
Narg2	34.600	45.350	20.400	13.100	0.00365
Nckap5	23.649	35.108	14.514	14.351	0.00740
Ncoa7	35.526	51.895	19.421	23.684	0.01521
Ncs1	30.667	48.067	16.733	17.867	0.02694
Necap1	32.964	41.179	17.893	18.357	0.00735
Nell2	53.769	60.615	24.538	30.462	0.09121
Nhsl1	31.500	47.500	17.500	20.167	0.00822
Nmnat2	30.107	43.143	17.036	17.500	0.00394
Nmt2, Rpp38, Acbd7, Olah	27.160	51.600	19.040	17.520	0.00901
Nol11	27.400	42.550	14.300	16.700	0.01533
Nr1h4	28.553	35.342	15.737	15.368	0.00555
Nrd1	50.222	77.407	27.185	28.407	0.00009
Nrg2	22.433	40.100	14.733	13.400	0.01676
Nuf2	32.947	55.684	22.632	20.474	0.00695
Nup153	27.895	39.368	16.158	14.947	0.09463
Osbp	53.818	83.182	34.909	33.182	0.04198
Osbpl9	37.343	63.457	22.057	24.000	0.00001
Pak6	53.312	49.812	23.625	21.125	0.00441

Pank3	45.000	69.154	26.308	17.154	0.00377
Papss1	46.824	64.823	30.941	24.706	0.01332
Pard3b	40.143	58.071	22.143	22.214	0.01579
Parn	20.583	31.667	9.042	14.708	0.03171
Parn	26.790	45.316	14.316	20.632	0.10476
Patl2, B2m, Trim69	33.375	50.375	18.583	18.333	0.00566
Pcf11	26.150	39.450	17.100	13.950	0.02066
Pcm1	42.474	68.210	22.579	25.368	0.00723
Pdgfd	28.875	35.417	16.792	15.208	0.03606
Pdzd2, Gm21706	37.538	61.077	21.692	24.692	0.02197
Pgc	38.913	58.696	22.391	23.174	0.00385
Pgk1	17.500	38.893	12.143	11.929	0.00198
Pip5k1a, Vps72	41.235	61.647	21.235	24.412	0.00524
Pkhd1l1	27.963	51.556	17.407	19.037	0.00768
Pkhd1l1	32.391	48.783	18.196	21.674	0.00002
Plxna4	31.444	54.222	18.111	21.944	0.00765
Poc1b	29.840	42.500	16.720	17.940	0.00009
Pot1a	35.231	59.308	16.615	26.308	0.02483
Ppp1cc	45.643	66.571	23.786	28.571	0.02661
Ppp1r12c, Tnnt1	36.889	65.778	26.963	23.037	0.01146
Ppp1r15b	48.870	77.696	31.652	29.000	0.00144
Prkab2	28.621	44.207	15.897	18.690	0.02910
Prnp	47.312	65.125	25.250	29.125	0.00998
Prr14l, Depdc5	47.231	78.769	27.923	23.385	0.00449
Prr14l, Depdc5	27.611	43.056	15.222	16.778	0.01499
Prr14l, Depdc5	45.905	73.952	21.857	32.952	0.00937
Prx	26.640	43.400	14.040	20.760	0.04198
Rab1	44.929	63.357	22.714	27.571	0.00967
Rab2b	26.000	44.875	19.188	15.625	0.12485
Rad51b	23.690	36.241	15.690	13.655	0.02692
Rasal2	23.667	36.952	15.857	12.191	0.03198
Rbpms	44.343	60.343	24.086	27.657	0.00200
Rfwd2	66.409	79.136	37.773	34.864	0.00409
Rgl1	27.750	38.150	15.550	14.700	0.07520
Rnf39, Ppp1r11, Znrd1, Znrd1as	24.529	47.176	18.529	15.412	0.03728
Rnu5g	55.950	80.400	30.200	27.350	0.00003
Rny3, Rny1	39.020	52.320	23.020	21.380	0.00000
Rock1	38.522	60.696	22.696	25.174	0.02041
Rps17, Cpeb1	39.167	56.583	16.167	25.167	0.04806
Rtdr1, Rab36	44.154	56.154	23.462	26.231	0.07083
Sec22a	54.059	70.882	29.588	32.471	0.00527
Sema3e	37.895	66.158	21.053	26.105	0.00538
Sepp1, Ccdc152	56.781	81.366	32.024	34.805	0.00000

Sesn1	37.182	68.364	24.636	24.364	0.03569
Sh2b1, Tufm, Atxn2l	28.722	45.833	13.556	13.778	0.00340
Sh3rf1, 1700001D01Rik	29.583	59.000	20.042	21.458	0.01232
Shb	48.500	79.750	31.750	30.000	0.04062
Slc10a6	43.821	70.857	30.821	26.071	0.00133
Slc1a3	23.652	38.783	16.348	13.913	0.01357
Slc25a30	35.632	50.684	16.210	19.790	0.00525
Slc48a1	41.500	62.019	25.111	25.815	0.00000
Slfn14-ps	57.292	82.750	34.125	32.792	0.00027
Slitrk3, Gm20754	21.304	36.348	12.348	14.652	0.01861
Smim13	27.062	44.125	15.938	19.312	0.13000
Sorbs1	63.273	77.818	30.091	31.546	0.01077
Sox12, Zcchc3, 6820408C15Rik	44.053	55.316	25.474	23.474	0.00760
Sp1	35.947	51.263	20.579	22.895	0.01229
Sp100	28.393	47.214	17.643	18.107	0.00556
Spag9	35.053	52.447	22.947	20.342	0.00049
Spcs3	40.706	59.118	22.177	24.588	0.02141
Spon2	36.000	58.706	23.765	21.647	0.01501
Sqstm1	31.750	57.167	17.750	25.583	0.06617
St3gal1, LOC101055818	37.095	51.048	19.095	24.143	0.03678
St3gal6	28.818	45.045	18.318	16.682	0.05111
Stard13	46.462	63.000	29.154	25.231	0.03293
Stard3nl	36.370	52.407	20.333	21.815	0.00752
Stard4	38.130	62.217	23.261	24.696	0.00878
Stat3	33.176	50.059	19.882	20.941	0.03634
Sulf1	25.286	37.952	15.286	15.000	0.10902
Synpo2	22.636	36.818	13.318	11.818	0.00195
Tacc1	40.421	53.789	20.421	23.105	0.00739
Tada2a	43.385	58.308	27.692	18.308	0.09008
Taok2, Tmem219	33.696	41.826	14.478	22.956	0.06385
Tceanc	38.781	50.156	22.625	19.875	0.00200
Tceanc	22.630	28.667	12.185	13.333	0.05225
Tcf4	36.167	65.333	23.500	22.417	0.02396
Tecr	66.941	91.059	39.529	36.941	0.00352
Tex14, Rnu1a1	61.935	88.936	33.903	35.419	0.00001
Tfdp2	28.462	55.154	19.077	22.154	0.18939
Tgfbr3	45.109	63.418	26.491	26.527	0.00000
Tjp2	35.412	58.824	21.765	24.588	0.07129
Tmem107, Snord118	45.857	72.929	28.143	31.179	0.00059
Tmem231	58.846	77.538	31.692	29.308	0.01358
Tmem41b	41.143	54.286	22.762	24.667	0.02174
Tmem44	32.125	50.542	16.667	19.500	0.00637
Tns1	39.571	53.714	23.000	19.143	0.07627

Tns4	49.889	70.667	28.444	30.056	0.00442
Tns4	31.840	38.200	15.560	19.360	0.09585
Trdmt1, Gm9875	38.895	50.316	19.421	18.895	0.02623
Trim24	43.333	64.333	24.600	20.400	0.00889
Trim8	53.583	69.583	24.750	29.500	0.05683
Tspan32	40.136	50.364	19.273	22.864	0.00330
Ttc30a2, Ttc30a1, Pde11a	30.526	58.211	20.895	22.316	0.01021
Tulp4	28.500	45.000	14.938	20.188	0.08542
Uba1, Cdk16	28.069	39.586	18.586	15.000	0.01748
Ubald1, Mgrn1	35.895	52.421	19.105	23.368	0.01596
Ube3a	40.214	65.821	32.464	20.464	0.00386
Ube3c	51.316	56.158	24.895	27.474	0.01259
Ubqln2	23.125	34.833	14.542	11.375	0.02491
Uck2	36.571	60.214	24.286	21.929	0.13652
Unc13b, Atp8b5	40.963	63.185	26.148	24.296	0.00363
Uqcc	28.177	45.294	21.823	13.294	0.08502
Usp18	45.958	65.375	23.875	28.042	0.00197
Usp49	41.125	49.438	19.062	24.250	0.04688
Usp53	29.591	46.682	15.954	16.909	0.00245
Vamp2, Per1	40.333	60.083	23.417	25.500	0.03993
Vegfa	45.929	68.857	24.857	26.929	0.02164
Vmp1	21.478	32.783	8.435	13.652	0.00772
Vmp1	23.625	38.708	13.375	17.500	0.08267
Vps13d	28.825	38.075	16.550	16.475	0.00461
Wdr70	25.902	38.317	17.195	14.756	0.00480
Wwox	21.150	44.300	15.900	11.550	0.01241
Yipf5	46.105	65.842	24.947	29.790	0.00955
Zfp13, Zscan10	42.171	65.286	27.057	25.914	0.00022
Zfp182, Spaca5	28.324	41.135	13.703	18.297	0.00085
Zfp184	35.800	51.133	20.933	19.533	0.01975
Zfp217	30.500	38.700	16.000	15.800	0.08391
Zfp219, Tmem253	26.421	42.421	16.158	15.210	0.04241
Zfp300	28.089	38.267	17.000	15.978	0.00064
Zfp341, Chmp4b	48.529	76.000	25.000	30.059	0.01538
Zfp366	28.474	38.053	13.368	18.158	0.05164
Zfp384, Ing4	27.357	60.071	14.571	17.857	0.00979
Zfp386	32.769	72.923	29.462	18.000	0.06474
Zfp474, 1700034E13Rik	38.250	45.125	18.812	19.312	0.03885
Zfp521	20.632	37.158	15.421	12.684	0.03585
Zfp566, Zfp260	31.954	47.727	15.727	20.318	0.02517
Zfr	24.125	37.125	14.542	14.458	0.08644
Zfyve1	35.840	59.720	21.560	23.080	0.01284
Zmiz2	32.227	54.955	22.546	18.682	0.00468

Zmym3, Nono	32.400	41.080	20.600	13.760	0.01208
Znrf1	59.455	70.636	26.727	33.545	0.03484

Table S4: List of genes showing downregulated H3K9Ac levels at Transcription Start Sites in MCEC treated with db/db EPC-EVs of in H3K9ac-CHIP-seq analysis.

Gene List	db+ EPC-1	db+ EPC-2	dbdb EPC-1	dbdb EPC-2	pvalue
0610030E20Rik, Tmem150a, Rnf181, Vamp5	14.216	8.622	24.703	25.459	0.02376
1700123I01Rik, Gpha2, Ppp2r5b	12.030	8.455	23.182	22.849	0.00026
2610507I01Rik, Mrpl55, 2310033P09Rik, Arf1	13.615	8.846	27.269	28.192	0.00000
4930481A15Rik, Drap1, AI837181	16.438	11.219	26.562	29.281	0.00000
9130023H24Rik, Armc5, Tgfb1i1	10.293	8.171	21.098	18.024	0.00000
AA543186, Man1b1	17.355	9.710	28.032	28.258	0.00005
Ak2	18.136	11.682	36.864	29.046	0.00010
Ankrd13b, Git1, Trp53i13	13.444	7.250	18.444	23.750	0.00007
Atat1, Mrps18b, Ppp1r10, Mir1894	15.954	6.953	24.977	26.000	0.06691
Azi1, Enthd2, 1810043H04Rik, Slc38a10	12.613	7.161	24.290	21.742	0.00000
B3gnt1, Brms1, Rin1	18.227	10.568	31.954	29.750	0.01937
Baiap2	13.718	6.949	24.128	25.359	0.00623
BC033916, BC051226, Daxx, Zbtb22, Gm19412, Tapbp	17.216	9.081	26.514	26.919	0.02400
Bcl6b, Mir497, Mir195, 0610010K14Rik, Rnasek, Alox12	9.353	5.088	15.971	23.059	0.00004
Bcl7c, Mir762, Ctf1	14.414	8.552	21.069	26.069	0.00004
Bop1, Hsf1	14.848	9.364	22.818	26.454	0.00002
Brd8, Kif20a, Cdc23	13.977	7.744	22.628	23.767	0.02296
Bsg, Hcn2	16.273	10.273	27.939	32.455	0.00000
Calm3	16.867	11.033	29.100	34.567	0.00000
Cant1	19.095	8.833	26.024	30.452	0.04400
Ccdc137, Arl16, Hgs	18.081	11.135	32.811	28.919	0.04941
Ccnk, Ccdc85c	11.200	6.575	20.750	16.575	0.00004
Ccs, Ccdc87	21.821	9.786	31.607	32.679	0.04847
Cd248, Tmem151a, Yif1a, Cnih2	24.718	13.177	38.306	41.153	0.02331
Cd2bp2, Tbc1d10b	12.735	5.928	25.241	19.422	0.03510
Cdca4	14.432	12.136	26.296	30.136	0.00000
Cebpz, Ndufaf7	15.871	9.226	33.903	31.032	0.00180
Cep170b	11.261	7.391	17.283	21.435	0.00000
Chtf18, Rpusd1, Mslnl	19.868	9.019	31.509	32.792	0.05748
Cirbp, 1600002K03Rik, Efna2	16.727	10.886	27.341	28.954	0.00459
Cnot10	12.794	8.000	20.235	22.147	0.00007
Cog2	15.688	8.542	28.458	30.521	0.02594
Cpne9, Brpf1	13.756	8.133	27.378	27.556	0.04347
Crtc1	15.269	7.769	28.808	21.000	0.05868
Csk	18.948	11.948	32.740	32.104	0.01494
Ctdnep1, Gabarap, Phf23, Dvl2	13.897	9.931	21.897	27.000	0.00014
Cyhr1, Kifc2, Foxh1, Ppp1r16a	14.450	8.712	25.633	26.383	0.01981
D030047H15Rik, Leng8, Leng9, Cdc42ep5	9.143	7.786	15.214	20.333	0.00001
D10Jhu81e, Pwp2	10.793	8.017	20.207	18.690	0.00000
D17H6S53E, Apom, Bag6, Prrc2a	13.816	10.868	22.684	28.316	0.00000
Dffb, Cep104	13.087	6.652	22.435	21.435	0.03386
Dnajc7, Nkiras2, Zfp385c	20.873	13.891	31.836	38.600	0.01470
Doc2g, Ndufv1	16.467	10.333	26.756	29.356	0.02710
Dot1l, Plekhj1, Sf3a2, Amh, Jsrp1	10.115	6.808	18.635	17.538	0.00001
Ehbp1l1, Fam89b, Sssca1	19.194	11.556	29.694	35.417	0.00656
Fads1, LOC101055817, Fen1, Tmem258, Myrf	21.742	15.161	38.903	36.677	0.00661

Fads1, LOC101055817, Fen1, Tmem258, Myrf	20.914	11.257	32.457	37.171	0.01787
Fam222b, Traf4, Nek8	14.191	6.404	26.425	21.425	0.05001
Fam73b, Dolpp1, Crat	13.723	6.362	22.575	26.575	0.01523
Fance	19.467	10.867	28.733	32.733	0.01253
Fasn, Ccdc57	13.353	8.647	23.206	25.647	0.00004
Fbxl16, Wdr24, Jmjd8, Stub1, Rhbdl1	15.657	5.514	25.057	29.257	0.07768
Fbxl18	13.762	9.000	30.071	31.381	0.00545
Fbxl18, D430018E03Rik	16.226	9.925	31.076	27.849	0.00746
Fem1a, Ticam1	18.891	11.435	31.370	32.109	0.01406
Foxk1	21.304	12.443	37.937	38.215	0.00395
Fzr1, Dohh, 2210404O07Rik, Nfic	15.062	7.979	24.896	23.458	0.05521
Galt, Il11ra1, Ccl27a	11.680	4.960	15.920	22.940	0.05083
Gga1	17.620	10.422	26.451	30.422	0.02222
Gga1, Gm10866, Sh3bp1	12.000	8.976	23.048	21.786	0.00001
Gins4, Golga7	12.528	8.694	24.278	24.639	0.00000
Gjc2, Guk1	10.550	6.750	21.500	19.525	0.00000
Gm4532, Zfp553	11.643	7.810	19.357	20.905	0.00033
Gpatch3, Gpn2	14.861	7.581	24.163	23.256	0.01267
Gpbar1, Aamp, Pnkd, Tmbim1	12.917	8.194	22.306	25.694	0.00000
Grik5, Zfp574	13.974	6.789	22.316	25.974	0.03611
Grin3b, Tmem259, Cnn2	15.773	10.796	30.114	27.909	0.00407
Gse1, Gins2, Gm10614	12.641	6.026	22.282	24.974	0.02440
Hras1, Lrrc56	10.268	7.439	23.634	24.024	0.00000
Ift172, Fndc4, Gckr	12.500	7.235	19.177	22.853	0.00119
Inpp5b, Mtf1	11.517	6.310	25.862	24.862	0.01585
Ints1	14.667	6.857	26.429	33.238	0.01248
Jag2, Nudt14	12.486	10.784	25.027	23.784	0.00000
Kcnh3, Mcrs1, 1700120C14Rik	17.311	11.068	29.838	33.000	0.00354
Kcnh4, Hcrt, Ghdc	13.180	7.538	25.923	23.103	0.01010
Kcnk4, Gpr137, Bad	18.771	7.571	27.371	31.971	0.05120
Khsrp, Slc25a41	14.143	8.000	26.171	20.657	0.00011
Klc1, Xrcc3, Zfyve21	14.969	8.766	26.375	28.469	0.00362
Kri1, Cdkn2d, Ap1m2	15.407	5.963	21.963	27.815	0.07325
Lamb2, Usp19	13.917	5.625	22.458	23.854	0.02060
Limk1, Gm10369	17.235	10.897	28.691	28.529	0.00302
Lpcat1	11.853	7.265	22.971	19.235	0.00001
Lrrc8a	12.114	5.057	20.886	15.914	0.00088
Ly6g5b, Csnk2b, Gpank1, D17H6S53E, Apom	16.909	5.333	29.242	27.394	0.04172
Mafk, Tmem184a	19.231	9.904	32.846	30.423	0.03349
Map3k4, 4732491K20Rik	11.903	10.613	23.258	23.677	0.00003
Mbtps1, Hsdl1	13.216	7.027	23.243	24.541	0.00000
Mbtps1, Hsdl1, Dnaaf1	11.120	6.104	22.040	18.440	0.03706
Men1, Map4k2	16.067	8.400	27.100	27.067	0.01698

Mex3a, Mir1905	13.275	9.175	22.450	24.800	0.00000
Micall1, 1700088E04Rik, Polr2f	18.250	7.958	30.042	30.083	0.03096
Midn	18.393	10.071	31.750	36.357	0.01654
Mpv17l2, Ifi30, Pik3r2, 2010320M18Rik, Mast3	22.145	13.194	40.065	35.242	0.01010
Mrpl12, Gm16755, Slc25a10	9.778	8.694	21.444	21.083	0.00001
Mrto4, Emc1	11.567	6.433	18.933	23.700	0.00004
Mvp, Pagr1a, Prrt2, Maz, Kif22	15.262	9.310	22.881	26.548	0.01869
Myo9b, Use1, Ocel1, Nr2f6	14.515	10.667	27.697	27.030	0.00000
Naaladl1, Sac3d1, Snx15	16.217	11.652	33.391	25.739	0.00001
Naaladl1, Sac3d1, Snx15	13.923	10.615	27.769	29.846	0.00001
Naaladl1, Sac3d1, Snx15	15.100	5.433	25.733	27.733	0.06868
Narfl, Haghl	15.462	10.051	29.641	36.821	0.00159
Narfl, Haghl, Ccdc78, Fam173a, Metrn	10.800	6.550	22.425	22.150	0.00000
Nlk	15.542	7.667	24.833	30.542	0.00005
Parp10, Grina	10.278	5.912	17.639	22.806	0.00001
Pcbp4, Gpr62, Parp3, Rrp9	11.559	8.559	24.147	20.971	0.00002
Pcnxl3, Map3k11	21.956	6.911	32.289	34.711	0.08025
Pcx, Lrfn4, Rce1, Gm960	13.030	8.303	22.000	26.636	0.00005
Pigz, 0610012G03Rik, Ncbp2	11.073	6.171	19.122	22.146	0.01867
Plcxd1, Gtpbp6, Zfp605	11.595	6.703	23.892	20.297	0.02105
Plxnb2, Dennd6b	14.760	10.200	23.800	28.840	0.00029
Pold1, Nr1h2	17.034	10.602	29.068	28.025	0.00312
Ppard	10.964	8.643	21.286	25.714	0.00000
Ppil1, BC004004	20.694	9.714	30.224	31.776	0.04274
Ppp1r14b, Fkbp2, Vegfb, Dnajc4	16.750	12.375	30.750	27.708	0.00039
Ppp2r5b, Atg2a	20.636	11.970	33.030	38.485	0.01456
Ppp2r5d, Pex6	12.645	7.355	18.936	23.419	0.00003
Psmb11, Cdh24, Acin1	11.902	7.854	19.488	22.707	0.00001
Psmg3	15.381	8.762	35.333	29.381	0.00001
Ptpn6, Grcc10, Rnu7, Atn1	12.241	8.690	21.345	29.103	0.00001
Rab35, Ccdc64	13.068	6.159	23.159	21.364	0.01102
Rab3a, Mpv17l2, Ifi30, Pik3r2	21.727	14.273	37.091	37.212	0.00749
Rab3a, Mpv17l2, Ifi30, Pik3r2, 2010320M18Rik, Mast3	14.172	8.759	23.690	24.897	0.00002
Radil	12.773	10.909	22.364	31.682	0.00048
Ranbp3, Vmac, Ndufa11	12.220	6.240	23.560	20.760	0.01791
Rasa4, Polr2j, Lrwd1	19.750	11.821	33.643	32.607	0.00000
Rbfa, Txnl4a	13.966	8.621	22.517	26.862	0.00003
Rexo1, Klf16	12.490	7.510	20.388	20.694	0.00000
Rgl2, H2-Ke2, Wdr46, B3galt4, Rps18	17.546	5.303	25.121	23.849	0.13434
Rhbdl1, Rhot2, Wdr90	12.389	7.167	22.861	21.750	0.00002
Rpap3, Endou	15.100	8.100	24.533	27.367	0.00001
Rrp36, Klhdc3, Mea1, Ppp2r5d	18.288	10.865	32.096	31.385	0.01661
Rrp36, Klhdc3, Mea1, Ppp2r5d	11.652	6.561	21.606	23.121	0.00696

Rsph6a, Dmwd, Dmpk, Mir3100	11.154	8.308	19.051	22.128	0.00002
Sart1, D330050I16Rik, Tsga10ip	19.852	12.185	40.074	32.667	0.00308
Scaf1, Rras, Prr12	11.375	7.917	20.417	21.875	0.02143
Skiv2l, Nelfe, Cfb, C2	10.059	8.382	21.647	28.382	0.00000
Slc11a1, Ctdsp1, Mir26b	14.220	9.627	24.322	24.915	0.00199
Slc26a8, Mapk14	15.771	7.029	26.886	27.286	0.02578
Slc27a5, Zbtb45	13.353	9.176	24.559	23.206	0.00001
Slc35e1, Med26	13.219	9.688	24.844	22.719	0.00021
Smarcc2, Myl6, Myl6b, LOC100502705	10.244	7.537	18.366	19.244	0.00001
Snhg10, Scarna13, Mir3069, Glrx5	24.558	14.154	38.519	39.308	0.02176
Sra1, Apbb3, Slc35a4, E230025N22Rik	16.556	9.444	27.815	29.630	0.00021
Srebf1	16.697	11.333	31.000	28.727	0.03120
Stambp, Gm21284	10.677	5.645	21.839	27.581	0.00000
Stk19, Dom3z, Skiv2l	14.950	5.825	26.525	30.125	0.03576
Surf6, Med22, Rpl7a, Surf1, Surf2	15.706	8.000	23.667	25.294	0.01641
Taf5, Usmg5, Pdcd11	16.000	7.710	23.452	24.710	0.05076
Tapbp, Rgl2, H2-Ke2, Wdr46	11.000	5.114	22.000	20.543	0.04871
Tjp3, Pip5k1c	14.542	7.514	23.556	22.264	0.04706
Tmem151a, Yif1a, Cnih2, Rab1b	16.692	9.231	27.269	30.038	0.00000
Tmem223, Tmem179b, Taf6l, Polr2g	15.237	8.026	27.263	29.737	0.01439
Tmem95, Kctd11, Acap1	12.667	9.500	19.833	24.867	0.00061
Tmub2, Atxn7l3	12.277	7.851	21.106	20.553	0.00001
Tonsl, Cyhr1	12.591	9.500	24.182	32.091	0.00001
Trappc12, Tssc1	17.074	7.889	27.074	32.481	0.06353
Troap, C1ql4	19.095	10.063	28.302	33.968	0.03202
Ttpal	16.127	8.182	25.055	25.382	0.02631
Ttyh1, D030047H15Rik, Leng8	11.988	6.096	17.133	21.482	0.04132
Tulp3, LOC101055755, Rhno1, Foxm1	11.515	6.606	18.636	23.273	0.00037
Txn2, Foxred2	12.806	11.472	26.417	23.806	0.00000
Uggt1	18.600	9.760	25.680	31.320	0.00012
Usp49, Tomm6, Gm14872, Prickle4, Frs3	18.348	15.696	36.435	39.348	0.00000
Usp5, Cdca3, Gnb3, Leprel2	12.200	9.400	22.689	24.956	0.00000
Vegfb, Dnajc4, Nudt22, Trpt1, Fermt3	12.963	5.519	25.074	28.074	0.01263
Wdr82, Ppm1m, Twf2	14.794	7.912	25.500	23.029	0.00000
Xab2, Pet100, Pcp2, Stxbp2	15.925	9.275	30.725	29.450	0.02876
Xpo5, Polr1c, Yipf3, Lrrc73	13.800	8.800	22.633	28.033	0.00001
Zbtb17	17.064	7.355	28.936	25.710	0.03616
Zc3h3	10.750	6.179	25.607	27.500	0.00000
Zfpm1	15.510	9.204	24.326	26.816	0.01874

Table S5: List of genes showing upregulated H3K9Ac levels at Transcription Start Sites in MCEC treated with db/db EPC-EVs of in H3K9ac-CHIP-seq analysis.
